# Supplementary material for: Genome-wide identification, characterization and gene expression of BES1 transcription factor family in grapevine (Vitis vinifera L.)
Source: Sci Rep. 2023 Jan 5;13:240. doi: 10.1038/s41598-022-24407-y (PMC9816167; doi:10.1038/s41598-022-24407-y)
Supplement: Supplementary file 3 — Supplementary Information. [file 41598_2022_24407_MOESM3_ESM.zip › Vvi_Vvi/Vitis_vinifera.PN40024.v4.dna_sm.toplevel.fa.vs.Vitis_vinifera.PN40024.v4.dna_sm.toplevel.fa.html/19.html]

|  |  |  |  |  |  |  |  |  |  |  |  |  |  |
| --- | --- | --- | --- | --- | --- | --- | --- | --- | --- | --- | --- | --- | --- |
| Duplication depth | Reference chromosome | Collinear blocks | | | | | | | | | | | |
| 0 | Vitvi19g01159\_t001 |  |  |  |  |  |  |  |  |  |  |  |  |
| 0 | Vitvi19g04000\_t001 |  |  |  |  |  |  |  |  |  |  |  |  |
| 0 | Vitvi19g04001\_t001 |  |  |  |  |  |  |  |  |  |  |  |  |
| 0 | Vitvi19g01126\_t001 |  |  |  |  |  |  |  |  |  |  |  |  |
| 0 | Vitvi19g04002\_t001 |  |  |  |  |  |  |  |  |  |  |  |  |
| 0 | Vitvi19g04003\_t001 |  |  |  |  |  |  |  |  |  |  |  |  |
| 0 | Vitvi19g02169\_t001 |  |  |  |  |  |  |  |  |  |  |  |  |
| 0 | Vitvi19g01079\_t001 |  |  |  |  |  |  |  |  |  |  |  |  |
| 0 | Vitvi19g04004\_t001 |  |  |  |  |  |  |  |  |  |  |  |  |
| 0 | Vitvi19g04005\_t001 |  |  |  |  |  |  |  |  |  |  |  |  |
| 0 | Vitvi19g04006\_t001 |  |  |  |  |  |  |  |  |  |  |  |  |
| 0 | Vitvi19g00001\_t001 |  |  |  |  |  |  |  |  |  |  |  |  |
| 0 | Vitvi19g00002\_t001 |  |  |  |  |  |  |  |  |  |  |  |  |
| 0 | Vitvi19g04007\_t001 |  |  |  |  |  |  |  |  |  |  |  |  |
| 0 | Vitvi19g00003\_t001 |  |  |  |  |  |  |  |  |  |  |  |  |
| 0 | Vitvi19g00005\_t001 |  |  |  |  |  |  |  |  |  |  |  |  |
| 0 | Vitvi19g00007\_t001 |  |  |  |  |  |  |  |  |  |  |  |  |
| 0 | Vitvi19g00008\_t002 |  |  |  |  |  |  |  |  |  |  |  |  |
| 0 | Vitvi19g00009\_t001 |  |  |  |  |  |  |  |  |  |  |  |  |
| 0 | Vitvi19g00010\_t002 |  |  |  |  |  |  |  |  |  |  |  |  |
| 0 | Vitvi19g00012\_t003 |  |  |  |  |  |  |  |  |  |  |  |  |
| 0 | Vitvi19g00013\_t001 |  |  |  |  |  |  |  |  |  |  |  |  |
| 0 | Vitvi19g00014\_t001 |  |  |  |  |  |  |  |  |  |  |  |  |
| 0 | Vitvi19g01793\_t001 |  |  |  |  |  |  |  |  |  |  |  |  |
| 0 | Vitvi19g00015\_t001 |  |  |  |  |  |  |  |  |  |  |  |  |
| 0 | Vitvi19g00016\_t001 |  |  |  |  |  |  |  |  |  |  |  |  |
| 0 | Vitvi19g00017\_t001 |  |  |  |  |  |  |  |  |  |  |  |  |
| 0 | Vitvi19g00018\_t001 |  |  |  |  |  |  |  |  |  |  |  |  |
| 0 | Vitvi19g04008\_t001 |  |  |  |  |  |  |  |  |  |  |  |  |
| 0 | Vitvi19g04009\_t001 |  |  |  |  |  |  |  |  |  |  |  |  |
| 0 | Vitvi19g00020\_t001 |  |  |  |  |  |  |  |  |  |  |  |  |
| 0 | Vitvi19g00021\_t001 |  |  |  |  |  |  |  |  |  |  |  |  |
| 0 | Vitvi19g00022\_t001 |  |  |  |  |  |  |  |  |  |  |  |  |
| 0 | Vitvi19g01797\_t001 |  |  |  |  |  |  |  |  |  |  |  |  |
| 0 | Vitvi19g00023\_t001 |  |  |  |  |  |  |  |  |  |  |  |  |
| 0 | Vitvi19g01798\_t001 |  |  |  |  |  |  |  |  |  |  |  |  |
| 0 | Vitvi19g00024\_t001 |  |  |  |  |  |  |  |  |  |  |  |  |
| 0 | Vitvi19g00025\_t002 |  |  |  |  |  |  |  |  |  |  |  |  |
| 0 | Vitvi19g00026\_t001 |  |  |  |  |  |  |  |  |  |  |  |  |
| 0 | Vitvi19g04010\_t001 |  |  |  |  |  |  |  |  |  |  |  |  |
| 0 | Vitvi19g04011\_t001 |  |  |  |  |  |  |  |  |  |  |  |  |
| 0 | Vitvi19g04012\_t001 |  |  |  |  |  |  |  |  |  |  |  |  |
| 0 | Vitvi19g00029\_t001 |  |  |  |  |  |  |  |  |  |  |  |  |
| 0 | Vitvi19g00030\_t001 |  |  |  |  |  |  |  |  |  |  |  |  |
| 0 | Vitvi19g00031\_t001 |  |  |  |  |  |  |  |  |  |  |  |  |
| 0 | Vitvi19g00034\_t001 |  |  |  |  |  |  |  |  |  |  |  |  |
| 0 | Vitvi19g00035\_t001 |  |  |  |  |  |  |  |  |  |  |  |  |
| 0 | Vitvi19g00036\_t001 |  |  |  |  |  |  |  |  |  |  |  |  |
| 0 | Vitvi19g00038\_t001 |  |  |  |  |  |  |  |  |  |  |  |  |
| 0 | Vitvi19g00041\_t001 |  |  |  |  |  |  |  |  |  |  |  |  |
| 0 | Vitvi19g04013\_t001 |  |  |  |  |  |  |  |  |  |  |  |  |
| 0 | Vitvi19g00042\_t001 |  |  |  |  |  |  |  |  |  |  |  |  |
| 0 | Vitvi19g01801\_t001 |  |  |  |  |  |  |  |  |  |  |  |  |
| 0 | Vitvi19g04014\_t001 |  |  |  |  |  |  |  |  |  |  |  |  |
| 0 | Vitvi19g04015\_t001 |  |  |  |  |  |  |  |  |  |  |  |  |
| 0 | Vitvi19g01805\_t001 |  |  |  |  |  |  |  |  |  |  |  |  |
| 0 | Vitvi19g04016\_t001 |  |  |  |  |  |  |  |  |  |  |  |  |
| 0 | Vitvi19g04017\_t001 |  |  |  |  |  |  |  |  |  |  |  |  |
| 0 | Vitvi19g04018\_t001 |  |  |  |  |  |  |  |  |  |  |  |  |
| 0 | Vitvi19g04019\_t001 |  |  |  |  |  |  |  |  |  |  |  |  |
| 0 | Vitvi19g00043\_t001 |  |  |  |  |  |  |  |  |  |  |  |  |
| 0 | Vitvi19g04020\_t001 |  |  |  |  |  |  |  |  |  |  |  |  |
| 0 | Vitvi19g00045\_t001 |  |  |  |  |  |  |  |  |  |  |  |  |
| 0 | Vitvi19g04021\_t002 |  |  |  |  |  |  |  |  |  |  |  |  |
| 0 | Vitvi19g04022\_t001 |  |  |  |  |  |  |  |  |  |  |  |  |
| 0 | Vitvi19g04023\_t001 |  |  |  |  |  |  |  |  |  |  |  |  |
| 0 | Vitvi19g04024\_t001 |  |  |  |  |  |  |  |  |  |  |  |  |
| 0 | Vitvi19g04025\_t001 |  |  |  |  |  |  |  |  |  |  |  |  |
| 0 | Vitvi19g00048\_t001 |  |  |  |  |  |  |  |  |  |  |  |  |
| 0 | Vitvi19g04026\_t001 |  |  |  |  |  |  |  |  |  |  |  |  |
| 1 | Vitvi19g04027\_t001 |  | Vitvi10g04382\_t001 |  |  |  |  |  |  |  |  |  |  |
| 1 | Vitvi19g01811\_t001 |  | \| \| |  |  |  |  |  |  |  |  |  |  |
| 1 | Vitvi19g01815\_t001 |  | \| \| |  |  |  |  |  |  |  |  |  |  |
| 1 | Vitvi19g04028\_t001 |  | \| \| |  |  |  |  |  |  |  |  |  |  |
| 1 | Vitvi19g04029\_t001 |  | \| \| |  |  |  |  |  |  |  |  |  |  |
| 1 | Vitvi19g00052\_t001 |  | \| \| |  |  |  |  |  |  |  |  |  |  |
| 1 | Vitvi19g04030\_t001 |  | \| \| |  |  |  |  |  |  |  |  |  |  |
| 1 | Vitvi19g01817\_t001 |  | \| \| |  |  |  |  |  |  |  |  |  |  |
| 1 | Vitvi19g04031\_t001 |  | \| \| |  |  |  |  |  |  |  |  |  |  |
| 1 | Vitvi19g01822\_t001 |  | \| \| |  |  |  |  |  |  |  |  |  |  |
| 1 | Vitvi19g04032\_t001 |  | \| \| |  |  |  |  |  |  |  |  |  |  |
| 1 | Vitvi19g01824\_t001 |  | \| \| |  |  |  |  |  |  |  |  |  |  |
| 1 | Vitvi19g01823\_t001 |  | \| \| |  |  |  |  |  |  |  |  |  |  |
| 1 | Vitvi19g01826\_t001 |  | \| \| |  |  |  |  |  |  |  |  |  |  |
| 1 | Vitvi19g00057\_t001 |  | \| \| |  |  |  |  |  |  |  |  |  |  |
| 1 | Vitvi19g00058\_t001 |  | Vitvi10g00640\_t001 |  |  |  |  |  |  |  |  |  |  |
| 1 | Vitvi19g00059\_t001 |  | \| \| |  |  |  |  |  |  |  |  |  |  |
| 1 | Vitvi19g00060\_t001 |  | Vitvi10g00637\_t002 |  |  |  |  |  |  |  |  |  |  |
| 1 | Vitvi19g00061\_t001 |  | Vitvi10g00636\_t001 |  |  |  |  |  |  |  |  |  |  |
| 1 | Vitvi19g04033\_t002 |  | \| \| |  |  |  |  |  |  |  |  |  |  |
| 1 | Vitvi19g04034\_t001 |  | \| \| |  |  |  |  |  |  |  |  |  |  |
| 1 | Vitvi19g04035\_t001 |  | \| \| |  |  |  |  |  |  |  |  |  |  |
| 1 | Vitvi19g04036\_t001 |  | \| \| |  |  |  |  |  |  |  |  |  |  |
| 1 | Vitvi19g00064\_t001 |  | \| \| |  |  |  |  |  |  |  |  |  |  |
| 1 | Vitvi19g04037\_t001 |  | \| \| |  |  |  |  |  |  |  |  |  |  |
| 1 | Vitvi19g04038\_t001 |  | \| \| |  |  |  |  |  |  |  |  |  |  |
| 1 | Vitvi19g00065\_t001 |  | \| \| |  |  |  |  |  |  |  |  |  |  |
| 1 | Vitvi19g00066\_t001 |  | \| \| |  |  |  |  |  |  |  |  |  |  |
| 1 | Vitvi19g04039\_t001 |  | \| \| |  |  |  |  |  |  |  |  |  |  |
| 1 | Vitvi19g04040\_t001 |  | \| \| |  |  |  |  |  |  |  |  |  |  |
| 1 | Vitvi19g00068\_t001 |  | \| \| |  |  |  |  |  |  |  |  |  |  |
| 1 | Vitvi19g00069\_t001 |  | \| \| |  |  |  |  |  |  |  |  |  |  |
| 1 | Vitvi19g04041\_t001 |  | \| \| |  |  |  |  |  |  |  |  |  |  |
| 1 | Vitvi19g00070\_t001 |  | \| \| |  |  |  |  |  |  |  |  |  |  |
| 1 | Vitvi19g00071\_t001 |  | \| \| |  |  |  |  |  |  |  |  |  |  |
| 1 | Vitvi19g00073\_t001 |  | \| \| |  |  |  |  |  |  |  |  |  |  |
| 1 | Vitvi19g00074\_t001 |  | \| \| |  |  |  |  |  |  |  |  |  |  |
| 1 | Vitvi19g01829\_t001 |  | Vitvi10g00628\_t001 |  |  |  |  |  |  |  |  |  |  |
| 1 | Vitvi19g00075\_t002 |  | \| \| |  |  |  |  |  |  |  |  |  |  |
| 1 | Vitvi19g00076\_t001 |  | \| \| |  |  |  |  |  |  |  |  |  |  |
| 1 | Vitvi19g04042\_t001 |  | \| \| |  |  |  |  |  |  |  |  |  |  |
| 1 | Vitvi19g01830\_t002 |  | Vitvi10g00626\_t001 |  |  |  |  |  |  |  |  |  |  |
| 1 | Vitvi19g00081\_t001 |  | \| \| |  |  |  |  |  |  |  |  |  |  |
| 1 | Vitvi19g00082\_t001 |  | \| \| |  |  |  |  |  |  |  |  |  |  |
| 1 | Vitvi19g00084\_t001 |  | \| \| |  |  |  |  |  |  |  |  |  |  |
| 1 | Vitvi19g00086\_t001 |  | \| \| |  |  |  |  |  |  |  |  |  |  |
| 1 | Vitvi19g04043\_t001 |  | \| \| |  |  |  |  |  |  |  |  |  |  |
| 1 | Vitvi19g04044\_t001 |  | \| \| |  |  |  |  |  |  |  |  |  |  |
| 1 | Vitvi19g01831\_t001 |  | \| \| |  |  |  |  |  |  |  |  |  |  |
| 1 | Vitvi19g04045\_t001 |  | \| \| |  |  |  |  |  |  |  |  |  |  |
| 1 | Vitvi19g04046\_t001 |  | \| \| |  |  |  |  |  |  |  |  |  |  |
| 1 | Vitvi19g04047\_t001 |  | \| \| |  |  |  |  |  |  |  |  |  |  |
| 1 | Vitvi19g04048\_t001 |  | Vitvi10g01798\_t001 |  |  |  |  |  |  |  |  |  |  |
| 1 | Vitvi19g04049\_t001 |  | \| \| |  |  |  |  |  |  |  |  |  |  |
| 1 | Vitvi19g04050\_t001 |  | \| \| |  |  |  |  |  |  |  |  |  |  |
| 1 | Vitvi19g01832\_t001 |  | \| \| |  |  |  |  |  |  |  |  |  |  |
| 1 | Vitvi19g00089\_t001 |  | \| \| |  |  |  |  |  |  |  |  |  |  |
| 1 | Vitvi19g01833\_t001 |  | Vitvi10g04372\_t001 |  |  |  |  |  |  |  |  |  |  |
| 1 | Vitvi19g01835\_t001 |  | \| \| |  |  |  |  |  |  |  |  |  |  |
| 1 | Vitvi19g00091\_t001 |  | Vitvi10g00619\_t001 |  |  |  |  |  |  |  |  |  |  |
| 1 | Vitvi19g00093\_t001 |  | \| \| |  |  |  |  |  |  |  |  |  |  |
| 1 | Vitvi19g00094\_t001 |  | \| \| |  |  |  |  |  |  |  |  |  |  |
| 1 | Vitvi19g00095\_t002 |  | \| \| |  |  |  |  |  |  |  |  |  |  |
| 1 | Vitvi19g00096\_t001 |  | \| \| |  |  |  |  |  |  |  |  |  |  |
| 1 | Vitvi19g00097\_t001 |  | \| \| |  |  |  |  |  |  |  |  |  |  |
| 1 | Vitvi19g01837\_t001 |  | Vitvi10g00598\_t001 |  |  |  |  |  |  |  |  |  |  |
| 1 | Vitvi19g00098\_t001 |  | Vitvi10g00597\_t001 |  |  |  |  |  |  |  |  |  |  |
| 1 | Vitvi19g00099\_t001 |  | \| \| |  |  |  |  |  |  |  |  |  |  |
| 2 | Vitvi19g00100\_t001 |  | \| \| |  | Vitvi12g00170\_t001 |  |  |  |  |  |  |  |  |
| 2 | Vitvi19g00102\_t001 |  | \| \| |  | \| \| |  |  |  |  |  |  |  |  |
| 2 | Vitvi19g00103\_t001 |  | \| \| |  | \| \| |  |  |  |  |  |  |  |  |
| 2 | Vitvi19g00104\_t001 |  | \| \| |  | \| \| |  |  |  |  |  |  |  |  |
| 2 | Vitvi19g00105\_t001 |  | \| \| |  | \| \| |  |  |  |  |  |  |  |  |
| 2 | Vitvi19g00106\_t001 |  | \| \| |  | \| \| |  |  |  |  |  |  |  |  |
| 2 | Vitvi19g01838\_t001 |  | \| \| |  | \| \| |  |  |  |  |  |  |  |  |
| 2 | Vitvi19g00107\_t002 |  | \| \| |  | \| \| |  |  |  |  |  |  |  |  |
| 2 | Vitvi19g00108\_t001 |  | Vitvi10g00583\_t001 |  | Vitvi12g00183\_t001 |  |  |  |  |  |  |  |  |
| 2 | Vitvi19g00112\_t001 |  | \| \| |  | Vitvi12g00185\_t001 |  |  |  |  |  |  |  |  |
| 2 | Vitvi19g00113\_t001 |  | \| \| |  | \| \| |  |  |  |  |  |  |  |  |
| 2 | Vitvi19g00114\_t001 |  | \| \| |  | \| \| |  |  |  |  |  |  |  |  |
| 2 | Vitvi19g00115\_t001 |  | Vitvi10g00579\_t003 |  | Vitvi12g00187\_t001 |  |  |  |  |  |  |  |  |
| 2 | Vitvi19g00116\_t001 |  | \| \| |  | \| \| |  |  |  |  |  |  |  |  |
| 2 | Vitvi19g00118\_t001 |  | \| \| |  | \| \| |  |  |  |  |  |  |  |  |
| 2 | Vitvi19g00120\_t001 |  | \| \| |  | \| \| |  |  |  |  |  |  |  |  |
| 2 | Vitvi19g00121\_t003 |  | \| \| |  | Vitvi12g00190\_t001 |  |  |  |  |  |  |  |  |
| 2 | Vitvi19g00122\_t001 |  | \| \| |  | Vitvi12g00193\_t001 |  |  |  |  |  |  |  |  |
| 2 | Vitvi19g00123\_t001 |  | \| \| |  | \| \| |  |  |  |  |  |  |  |  |
| 2 | Vitvi19g00124\_t001 |  | \| \| |  | \| \| |  |  |  |  |  |  |  |  |
| 2 | Vitvi19g00125\_t001 |  | \| \| |  | \| \| |  |  |  |  |  |  |  |  |
| 2 | Vitvi19g00127\_t001 |  | \| \| |  | \| \| |  |  |  |  |  |  |  |  |
| 2 | Vitvi19g00128\_t001 |  | \| \| |  | Vitvi12g00201\_t001 |  |  |  |  |  |  |  |  |
| 2 | Vitvi19g00129\_t001 |  | \| \| |  | \| \| |  |  |  |  |  |  |  |  |
| 2 | Vitvi19g04051\_t001 |  | \| \| |  | \| \| |  |  |  |  |  |  |  |  |
| 2 | Vitvi19g00130\_t001 |  | \| \| |  | \| \| |  |  |  |  |  |  |  |  |
| 2 | Vitvi19g04052\_t001 |  | \| \| |  | \| \| |  |  |  |  |  |  |  |  |
| 2 | Vitvi19g00131\_t001 |  | Vitvi10g04360\_t001 |  | \| \| |  |  |  |  |  |  |  |  |
| 2 | Vitvi19g04053\_t001 |  | \| \| |  | \| \| |  |  |  |  |  |  |  |  |
| 2 | Vitvi19g00132\_t001 |  | \| \| |  | Vitvi12g00205\_t001 |  |  |  |  |  |  |  |  |
| 2 | Vitvi19g00134\_t001 |  | \| \| |  | \| \| |  |  |  |  |  |  |  |  |
| 2 | Vitvi19g00136\_t001 |  | \| \| |  | \| \| |  |  |  |  |  |  |  |  |
| 2 | Vitvi19g00137\_t001 |  | \| \| |  | \| \| |  |  |  |  |  |  |  |  |
| 2 | Vitvi19g00138\_t001 |  | Vitvi10g00554\_t001 |  | \| \| |  |  |  |  |  |  |  |  |
| 2 | Vitvi19g01842\_t001 |  | \| \| |  | Vitvi12g00215\_t003 |  |  |  |  |  |  |  |  |
| 2 | Vitvi19g04054\_t001 |  | \| \| |  | \| \| |  |  |  |  |  |  |  |  |
| 2 | Vitvi19g04055\_t001 |  | \| \| |  | \| \| |  |  |  |  |  |  |  |  |
| 2 | Vitvi19g04056\_t001 |  | \| \| |  | Vitvi12g04053\_t001 |  |  |  |  |  |  |  |  |
| 2 | Vitvi19g04057\_t001 |  | \| \| |  | \| \| |  |  |  |  |  |  |  |  |
| 2 | Vitvi19g00139\_t001 |  | \| \| |  | \| \| |  |  |  |  |  |  |  |  |
| 2 | Vitvi19g04058\_t001 |  | \| \| |  | \| \| |  |  |  |  |  |  |  |  |
| 2 | Vitvi19g00140\_t001 |  | Vitvi10g00540\_t001 |  | Vitvi12g00219\_t001 |  |  |  |  |  |  |  |  |
| 2 | Vitvi19g00141\_t001 |  | Vitvi10g00539\_t001 |  | \| \| |  |  |  |  |  |  |  |  |
| 2 | Vitvi19g04059\_t001 |  | \| \| |  | \| \| |  |  |  |  |  |  |  |  |
| 2 | Vitvi19g00142\_t001 |  | \| \| |  | \| \| |  |  |  |  |  |  |  |  |
| 2 | Vitvi19g01844\_t001 |  | Vitvi10g04345\_t001 |  | \| \| |  |  |  |  |  |  |  |  |
| 2 | Vitvi19g00143\_t001 |  | Vitvi10g00537\_t001 |  | Vitvi12g02265\_t001 |  |  |  |  |  |  |  |  |
| 2 | Vitvi19g00144\_t001 |  | \| \| |  | \| \| |  |  |  |  |  |  |  |  |
| 2 | Vitvi19g04060\_t001 |  | \| \| |  | \| \| |  |  |  |  |  |  |  |  |
| 2 | Vitvi19g04061\_t001 |  | \| \| |  | \| \| |  |  |  |  |  |  |  |  |
| 2 | Vitvi19g04062\_t001 |  | \| \| |  | \| \| |  |  |  |  |  |  |  |  |
| 2 | Vitvi19g00145\_t001 |  | \| \| |  | \| \| |  |  |  |  |  |  |  |  |
| 2 | Vitvi19g04063\_t001 |  | \| \| |  | \| \| |  |  |  |  |  |  |  |  |
| 2 | Vitvi19g04064\_t001 |  | \| \| |  | \| \| |  |  |  |  |  |  |  |  |
| 2 | Vitvi19g01847\_t001 |  | Vitvi10g01787\_t001 |  | \| \| |  |  |  |  |  |  |  |  |
| 2 | Vitvi19g01848\_t001 |  | \| \| |  | \| \| |  |  |  |  |  |  |  |  |
| 2 | Vitvi19g01849\_t001 |  | \| \| |  | \| \| |  |  |  |  |  |  |  |  |
| 2 | Vitvi19g00147\_t001 |  | \| \| |  | Vitvi12g00226\_t001 |  |  |  |  |  |  |  |  |
| 2 | Vitvi19g00148\_t001 |  | Vitvi10g00529\_t001 |  | \| \| |  |  |  |  |  |  |  |  |
| 2 | Vitvi19g00150\_t001 |  | \| \| |  | \| \| |  |  |  |  |  |  |  |  |
| 2 | Vitvi19g00151\_t003 |  | \| \| |  | \| \| |  |  |  |  |  |  |  |  |
| 2 | Vitvi19g00153\_t001 |  | Vitvi10g01781\_t001 |  | Vitvi12g00233\_t001 |  |  |  |  |  |  |  |  |
| 1 | Vitvi19g01850\_t001 |  | \| \| |  |  |  |  |  |  |  |  |  |  |
| 1 | Vitvi19g04065\_t001 |  | \| \| |  |  |  |  |  |  |  |  |  |  |
| 1 | Vitvi19g01851\_t001 |  | \| \| |  |  |  |  |  |  |  |  |  |  |
| 1 | Vitvi19g00155\_t003 |  | \| \| |  |  |  |  |  |  |  |  |  |  |
| 1 | Vitvi19g00156\_t001 |  | \| \| |  |  |  |  |  |  |  |  |  |  |
| 1 | Vitvi19g01852\_t002 |  | Vitvi10g04335\_t001 |  |  |  |  |  |  |  |  |  |  |
| 1 | Vitvi19g00157\_t001 |  | \| \| |  |  |  |  |  |  |  |  |  |  |
| 1 | Vitvi19g00158\_t001 |  | \| \| |  |  |  |  |  |  |  |  |  |  |
| 1 | Vitvi19g01853\_t001 |  | \| \| |  |  |  |  |  |  |  |  |  |  |
| 1 | Vitvi19g04066\_t001 |  | \| \| |  |  |  |  |  |  |  |  |  |  |
| 1 | Vitvi19g00159\_t001 |  | \| \| |  |  |  |  |  |  |  |  |  |  |
| 1 | Vitvi19g00161\_t001 |  | Vitvi10g00511\_t001 |  |  |  |  |  |  |  |  |  |  |
| 1 | Vitvi19g00162\_t001 |  | \| \| |  |  |  |  |  |  |  |  |  |  |
| 1 | Vitvi19g00164\_t001 |  | \| \| |  |  |  |  |  |  |  |  |  |  |
| 1 | Vitvi19g00165\_t001 |  | \| \| |  |  |  |  |  |  |  |  |  |  |
| 1 | Vitvi19g00166\_t001 |  | \| \| |  |  |  |  |  |  |  |  |  |  |
| 1 | Vitvi19g00167\_t001 |  | \| \| |  |  |  |  |  |  |  |  |  |  |
| 1 | Vitvi19g04067\_t001 |  | \| \| |  |  |  |  |  |  |  |  |  |  |
| 1 | Vitvi19g00170\_t001 |  | \| \| |  |  |  |  |  |  |  |  |  |  |
| 1 | Vitvi19g00171\_t001 |  | \| \| |  |  |  |  |  |  |  |  |  |  |
| 1 | Vitvi19g04068\_t001 |  | \| \| |  |  |  |  |  |  |  |  |  |  |
| 1 | Vitvi19g04069\_t001 |  | \| \| |  |  |  |  |  |  |  |  |  |  |
| 1 | Vitvi19g01854\_t001 |  | \| \| |  |  |  |  |  |  |  |  |  |  |
| 1 | Vitvi19g04070\_t001 |  | \| \| |  |  |  |  |  |  |  |  |  |  |
| 1 | Vitvi19g04071\_t001 |  | \| \| |  |  |  |  |  |  |  |  |  |  |
| 1 | Vitvi19g04072\_t001 |  | \| \| |  |  |  |  |  |  |  |  |  |  |
| 1 | Vitvi19g00174\_t001 |  | \| \| |  |  |  |  |  |  |  |  |  |  |
| 1 | Vitvi19g00175\_t001 |  | \| \| |  |  |  |  |  |  |  |  |  |  |
| 1 | Vitvi19g04073\_t001 |  | \| \| |  |  |  |  |  |  |  |  |  |  |
| 1 | Vitvi19g04074\_t001 |  | \| \| |  |  |  |  |  |  |  |  |  |  |
| 1 | Vitvi19g04075\_t001 |  | \| \| |  |  |  |  |  |  |  |  |  |  |
| 1 | Vitvi19g00177\_t001 |  | \| \| |  |  |  |  |  |  |  |  |  |  |
| 1 | Vitvi19g04076\_t001 |  | \| \| |  |  |  |  |  |  |  |  |  |  |
| 1 | Vitvi19g00178\_t001 |  | \| \| |  |  |  |  |  |  |  |  |  |  |
| 1 | Vitvi19g00179\_t001 |  | \| \| |  |  |  |  |  |  |  |  |  |  |
| 1 | Vitvi19g00180\_t001 |  | \| \| |  |  |  |  |  |  |  |  |  |  |
| 2 | Vitvi19g01855\_t001 |  | Vitvi10g01773\_t001 |  | Vitvi12g00253\_t001 |  |  |  |  |  |  |  |  |
| 2 | Vitvi19g00181\_t001 |  | \| \| |  | \| \| |  |  |  |  |  |  |  |  |
| 2 | Vitvi19g01856\_t001 |  | \| \| |  | \| \| |  |  |  |  |  |  |  |  |
| 2 | Vitvi19g00182\_t001 |  | \| \| |  | \| \| |  |  |  |  |  |  |  |  |
| 2 | Vitvi19g01857\_t001 |  | \| \| |  | \| \| |  |  |  |  |  |  |  |  |
| 2 | Vitvi19g00184\_t001 |  | Vitvi10g00491\_t001 |  | \| \| |  |  |  |  |  |  |  |  |
| 2 | Vitvi19g00185\_t001 |  | \| \| |  | \| \| |  |  |  |  |  |  |  |  |
| 2 | Vitvi19g04077\_t001 |  | \| \| |  | \| \| |  |  |  |  |  |  |  |  |
| 2 | Vitvi19g00186\_t001 |  | \| \| |  | Vitvi12g00270\_t001 |  |  |  |  |  |  |  |  |
| 2 | Vitvi19g00188\_t001 |  | \| \| |  | \| \| |  |  |  |  |  |  |  |  |
| 2 | Vitvi19g00189\_t001 |  | \| \| |  | \| \| |  |  |  |  |  |  |  |  |
| 2 | Vitvi19g04078\_t001 |  | \| \| |  | \| \| |  |  |  |  |  |  |  |  |
| 2 | Vitvi19g00190\_t001 |  | \| \| |  | \| \| |  |  |  |  |  |  |  |  |
| 2 | Vitvi19g04079\_t001 |  | \| \| |  | \| \| |  |  |  |  |  |  |  |  |
| 2 | Vitvi19g00191\_t001 |  | \| \| |  | \| \| |  |  |  |  |  |  |  |  |
| 2 | Vitvi19g01784\_t001 |  | Vitvi10g00488\_t001 |  | Vitvi12g00274\_t001 |  |  |  |  |  |  |  |  |
| 2 | Vitvi19g04080\_t001 |  | \| \| |  | \| \| |  |  |  |  |  |  |  |  |
| 2 | Vitvi19g00194\_t001 |  | \| \| |  | \| \| |  |  |  |  |  |  |  |  |
| 2 | Vitvi19g01858\_t003 |  | \| \| |  | \| \| |  |  |  |  |  |  |  |  |
| 2 | Vitvi19g00195\_t001 |  | \| \| |  | \| \| |  |  |  |  |  |  |  |  |
| 2 | Vitvi19g00196\_t001 |  | \| \| |  | \| \| |  |  |  |  |  |  |  |  |
| 2 | Vitvi19g00197\_t001 |  | \| \| |  | \| \| |  |  |  |  |  |  |  |  |
| 2 | Vitvi19g00198\_t001 |  | \| \| |  | \| \| |  |  |  |  |  |  |  |  |
| 2 | Vitvi19g01859\_t001 |  | \| \| |  | \| \| |  |  |  |  |  |  |  |  |
| 2 | Vitvi19g00199\_t001 |  | \| \| |  | Vitvi12g00277\_t001 |  |  |  |  |  |  |  |  |
| 2 | Vitvi19g00200\_t001 |  | Vitvi10g04328\_t002 |  | Vitvi12g00280\_t001 |  |  |  |  |  |  |  |  |
| 2 | Vitvi19g00201\_t001 |  | \| \| |  | \| \| |  |  |  |  |  |  |  |  |
| 2 | Vitvi19g04081\_t001 |  | \| \| |  | \| \| |  |  |  |  |  |  |  |  |
| 2 | Vitvi19g00202\_t001 |  | \| \| |  | \| \| |  |  |  |  |  |  |  |  |
| 2 | Vitvi19g00204\_t001 |  | \| \| |  | \| \| |  |  |  |  |  |  |  |  |
| 2 | Vitvi19g01862\_t001 |  | \| \| |  | \| \| |  |  |  |  |  |  |  |  |
| 2 | Vitvi19g04082\_t001 |  | \| \| |  | \| \| |  |  |  |  |  |  |  |  |
| 2 | Vitvi19g04083\_t001 |  | \| \| |  | \| \| |  |  |  |  |  |  |  |  |
| 2 | Vitvi19g00205\_t001 |  | \| \| |  | \| \| |  |  |  |  |  |  |  |  |
| 2 | Vitvi19g01864\_t001 |  | \| \| |  | \| \| |  |  |  |  |  |  |  |  |
| 2 | Vitvi19g04084\_t001 |  | \| \| |  | \| \| |  |  |  |  |  |  |  |  |
| 2 | Vitvi19g00206\_t001 |  | \| \| |  | Vitvi12g00290\_t001 |  |  |  |  |  |  |  |  |
| 2 | Vitvi19g01865\_t001 |  | Vitvi10g02258\_t001 |  | \| \| |  |  |  |  |  |  |  |  |
| 2 | Vitvi19g00207\_t004 |  | \| \| |  | \| \| |  |  |  |  |  |  |  |  |
| 2 | Vitvi19g00208\_t001 |  | \| \| |  | Vitvi12g04066\_t001 |  |  |  |  |  |  |  |  |
| 2 | Vitvi19g00209\_t001 |  | Vitvi10g02257\_t001 |  | \| \| |  |  |  |  |  |  |  |  |
| 2 | Vitvi19g00210\_t001 |  | \| \| |  | \| \| |  |  |  |  |  |  |  |  |
| 2 | Vitvi19g01867\_t001 |  | \| \| |  | \| \| |  |  |  |  |  |  |  |  |
| 2 | Vitvi19g00214\_t001 |  | \| \| |  | \| \| |  |  |  |  |  |  |  |  |
| 2 | Vitvi19g01790\_t001 |  | \| \| |  | \| \| |  |  |  |  |  |  |  |  |
| 2 | Vitvi19g04085\_t001 |  | \| \| |  | \| \| |  |  |  |  |  |  |  |  |
| 2 | Vitvi19g00217\_t001 |  | \| \| |  | \| \| |  |  |  |  |  |  |  |  |
| 2 | Vitvi19g00218\_t001 |  | \| \| |  | Vitvi12g00297\_t001 |  |  |  |  |  |  |  |  |
| 2 | Vitvi19g04086\_t001 |  | \| \| |  | \| \| |  |  |  |  |  |  |  |  |
| 2 | Vitvi19g00219\_t001 |  | \| \| |  | \| \| |  |  |  |  |  |  |  |  |
| 2 | Vitvi19g01869\_t001.1.6037826c |  | \| \| |  | \| \| |  |  |  |  |  |  |  |  |
| 2 | Vitvi19g00220\_t001 |  | \| \| |  | Vitvi12g00299\_t002 |  |  |  |  |  |  |  |  |
| 2 | Vitvi19g00221\_t001 |  | \| \| |  | Vitvi12g00301\_t001 |  |  |  |  |  |  |  |  |
| 2 | Vitvi19g00222\_t001 |  | \| \| |  | \| \| |  |  |  |  |  |  |  |  |
| 2 | Vitvi19g00223\_t001 |  | \| \| |  | \| \| |  |  |  |  |  |  |  |  |
| 2 | Vitvi19g00224\_t001 |  | \| \| |  | \| \| |  |  |  |  |  |  |  |  |
| 2 | Vitvi19g00225\_t001 |  | Vitvi10g00455\_t001 |  | \| \| |  |  |  |  |  |  |  |  |
| 2 | Vitvi19g04087\_t001 |  | \| \| |  | Vitvi12g00302\_t001 |  |  |  |  |  |  |  |  |
| 2 | Vitvi19g01871\_t001 |  | \| \| |  | \| \| |  |  |  |  |  |  |  |  |
| 2 | Vitvi19g00227\_t001 |  | \| \| |  | \| \| |  |  |  |  |  |  |  |  |
| 2 | Vitvi19g00228\_t001 |  | Vitvi10g01764\_t001 |  | Vitvi12g00304\_t001 |  |  |  |  |  |  |  |  |
| 2 | Vitvi19g04088\_t001 |  | \| \| |  | \| \| |  |  |  |  |  |  |  |  |
| 2 | Vitvi19g04089\_t001 |  | \| \| |  | \| \| |  |  |  |  |  |  |  |  |
| 2 | Vitvi19g01872\_t001 |  | Vitvi10g00461\_t001 |  | \| \| |  |  |  |  |  |  |  |  |
| 2 | Vitvi19g01873\_t001 |  | \| \| |  | \| \| |  |  |  |  |  |  |  |  |
| 2 | Vitvi19g01874\_t001 |  | \| \| |  | \| \| |  |  |  |  |  |  |  |  |
| 2 | Vitvi19g00229\_t001 |  | \| \| |  | \| \| |  |  |  |  |  |  |  |  |
| 2 | Vitvi19g01875\_t001 |  | \| \| |  | \| \| |  |  |  |  |  |  |  |  |
| 2 | Vitvi19g04090\_t001 |  | \| \| |  | \| \| |  |  |  |  |  |  |  |  |
| 2 | Vitvi19g01876\_t001 |  | \| \| |  | \| \| |  |  |  |  |  |  |  |  |
| 2 | Vitvi19g00231\_t001 |  | \| \| |  | \| \| |  |  |  |  |  |  |  |  |
| 2 | Vitvi19g00233\_t001 |  | \| \| |  | \| \| |  |  |  |  |  |  |  |  |
| 2 | Vitvi19g00234\_t001 |  | Vitvi10g04315\_t001 |  | \| \| |  |  |  |  |  |  |  |  |
| 2 | Vitvi19g00235\_t001 |  | \| \| |  | \| \| |  |  |  |  |  |  |  |  |
| 2 | Vitvi19g01878\_t004 |  | \| \| |  | \| \| |  |  |  |  |  |  |  |  |
| 2 | Vitvi19g01880\_t001 |  | \| \| |  | Vitvi12g00319\_t001 |  |  |  |  |  |  |  |  |
| 2 | Vitvi19g00239\_t001 |  | Vitvi10g00452\_t002 |  | Vitvi12g00320\_t001 |  |  |  |  |  |  |  |  |
| 2 | Vitvi19g00240\_t001 |  | \| \| |  | \| \| |  |  |  |  |  |  |  |  |
| 2 | Vitvi19g00241\_t001 |  | Vitvi10g00470\_t001 |  | Vitvi12g00322\_t001 |  |  |  |  |  |  |  |  |
| 1 | Vitvi19g01881\_t001 |  | \| \| |  |  |  |  |  |  |  |  |  |  |
| 1 | Vitvi19g01883\_t001 |  | \| \| |  |  |  |  |  |  |  |  |  |  |
| 1 | Vitvi19g01884\_t001 |  | \| \| |  |  |  |  |  |  |  |  |  |  |
| 1 | Vitvi19g00242\_t001 |  | \| \| |  |  |  |  |  |  |  |  |  |  |
| 1 | Vitvi19g04091\_t001 |  | \| \| |  |  |  |  |  |  |  |  |  |  |
| 1 | Vitvi19g04092\_t001 |  | \| \| |  |  |  |  |  |  |  |  |  |  |
| 1 | Vitvi19g00243\_t001 |  | \| \| |  |  |  |  |  |  |  |  |  |  |
| 1 | Vitvi19g00245\_t001 |  | \| \| |  |  |  |  |  |  |  |  |  |  |
| 1 | Vitvi19g00246\_t001 |  | \| \| |  |  |  |  |  |  |  |  |  |  |
| 1 | Vitvi19g04093\_t001 |  | \| \| |  |  |  |  |  |  |  |  |  |  |
| 1 | Vitvi19g04094\_t001 |  | \| \| |  |  |  |  |  |  |  |  |  |  |
| 1 | Vitvi19g04095\_t001 |  | \| \| |  |  |  |  |  |  |  |  |  |  |
| 1 | Vitvi19g04096\_t001 |  | \| \| |  |  |  |  |  |  |  |  |  |  |
| 1 | Vitvi19g01889\_t001 |  | \| \| |  |  |  |  |  |  |  |  |  |  |
| 1 | Vitvi19g04097\_t001 |  | \| \| |  |  |  |  |  |  |  |  |  |  |
| 1 | Vitvi19g04098\_t001 |  | \| \| |  |  |  |  |  |  |  |  |  |  |
| 1 | Vitvi19g04099\_t001 |  | \| \| |  |  |  |  |  |  |  |  |  |  |
| 1 | Vitvi19g04100\_t001 |  | \| \| |  |  |  |  |  |  |  |  |  |  |
| 1 | Vitvi19g04101\_t001 |  | \| \| |  |  |  |  |  |  |  |  |  |  |
| 1 | Vitvi19g04102\_t001 |  | \| \| |  |  |  |  |  |  |  |  |  |  |
| 1 | Vitvi19g04103\_t001 |  | \| \| |  |  |  |  |  |  |  |  |  |  |
| 1 | Vitvi19g01893\_t001 |  | \| \| |  |  |  |  |  |  |  |  |  |  |
| 1 | Vitvi19g00251\_t001 |  | \| \| |  |  |  |  |  |  |  |  |  |  |
| 1 | Vitvi19g00252\_t001 |  | Vitvi10g04302\_t001 |  |  |  |  |  |  |  |  |  |  |
| 1 | Vitvi19g04104\_t001 |  | \| \| |  |  |  |  |  |  |  |  |  |  |
| 1 | Vitvi19g00253\_t001 |  | \| \| |  |  |  |  |  |  |  |  |  |  |
| 1 | Vitvi19g00254\_t001 |  | \| \| |  |  |  |  |  |  |  |  |  |  |
| 1 | Vitvi19g00255\_t001 |  | Vitvi10g00451\_t001 |  |  |  |  |  |  |  |  |  |  |
| 1 | Vitvi19g00256\_t001 |  | \| \| |  |  |  |  |  |  |  |  |  |  |
| 1 | Vitvi19g00257\_t001 |  | \| \| |  |  |  |  |  |  |  |  |  |  |
| 1 | Vitvi19g00258\_t001 |  | \| \| |  |  |  |  |  |  |  |  |  |  |
| 1 | Vitvi19g00260\_t001 |  | \| \| |  |  |  |  |  |  |  |  |  |  |
| 1 | Vitvi19g00261\_t003 |  | Vitvi10g01762\_t001 |  |  |  |  |  |  |  |  |  |  |
| 1 | Vitvi19g01895\_t001 |  | Vitvi10g04287\_t001 |  |  |  |  |  |  |  |  |  |  |
| 1 | Vitvi19g00262\_t001 |  | \| \| |  |  |  |  |  |  |  |  |  |  |
| 1 | Vitvi19g01896\_t001 |  | \| \| |  |  |  |  |  |  |  |  |  |  |
| 1 | Vitvi19g00263\_t001 |  | \| \| |  |  |  |  |  |  |  |  |  |  |
| 1 | Vitvi19g00264\_t001 |  | \| \| |  |  |  |  |  |  |  |  |  |  |
| 1 | Vitvi19g00265\_t001 |  | \| \| |  |  |  |  |  |  |  |  |  |  |
| 1 | Vitvi19g04105\_t001 |  | \| \| |  |  |  |  |  |  |  |  |  |  |
| 1 | Vitvi19g04106\_t001 |  | \| \| |  |  |  |  |  |  |  |  |  |  |
| 1 | Vitvi19g00270\_t001 |  | Vitvi10g00437\_t001 |  |  |  |  |  |  |  |  |  |  |
| 1 | Vitvi19g00271\_t001 |  | \| \| |  |  |  |  |  |  |  |  |  |  |
| 1 | Vitvi19g01897\_t001 |  | \| \| |  |  |  |  |  |  |  |  |  |  |
| 1 | Vitvi19g00272\_t001 |  | \| \| |  |  |  |  |  |  |  |  |  |  |
| 1 | Vitvi19g04107\_t001 |  | \| \| |  |  |  |  |  |  |  |  |  |  |
| 1 | Vitvi19g00274\_t001 |  | \| \| |  |  |  |  |  |  |  |  |  |  |
| 1 | Vitvi19g04108\_t001 |  | \| \| |  |  |  |  |  |  |  |  |  |  |
| 1 | Vitvi19g00275\_t001 |  | \| \| |  |  |  |  |  |  |  |  |  |  |
| 1 | Vitvi19g00276\_t001 |  | Vitvi10g00398\_t001 |  |  |  |  |  |  |  |  |  |  |
| 1 | Vitvi19g04109\_t001 |  | \| \| |  |  |  |  |  |  |  |  |  |  |
| 1 | Vitvi19g00280\_t001 |  | \| \| |  |  |  |  |  |  |  |  |  |  |
| 1 | Vitvi19g00282\_t001 |  | Vitvi10g00406\_t001 |  |  |  |  |  |  |  |  |  |  |
| 1 | Vitvi19g00283\_t001 |  | \| \| |  |  |  |  |  |  |  |  |  |  |
| 1 | Vitvi19g04110\_t001 |  | \| \| |  |  |  |  |  |  |  |  |  |  |
| 1 | Vitvi19g01899\_t001 |  | \| \| |  |  |  |  |  |  |  |  |  |  |
| 1 | Vitvi19g04111\_t001 |  | \| \| |  |  |  |  |  |  |  |  |  |  |
| 1 | Vitvi19g01900\_t001 |  | \| \| |  |  |  |  |  |  |  |  |  |  |
| 1 | Vitvi19g00284\_t001 |  | \| \| |  |  |  |  |  |  |  |  |  |  |
| 1 | Vitvi19g00285\_t001 |  | \| \| |  |  |  |  |  |  |  |  |  |  |
| 1 | Vitvi19g00286\_t001 |  | Vitvi10g00394\_t001 |  |  |  |  |  |  |  |  |  |  |
| 1 | Vitvi19g01901\_t001.2.6037826c |  | \| \| |  |  |  |  |  |  |  |  |  |  |
| 1 | Vitvi19g01902\_t001 |  | \| \| |  |  |  |  |  |  |  |  |  |  |
| 1 | Vitvi19g04112\_t001 |  | \| \| |  |  |  |  |  |  |  |  |  |  |
| 1 | Vitvi19g00290\_t001 |  | Vitvi10g00391\_t001 |  |  |  |  |  |  |  |  |  |  |
| 1 | Vitvi19g00291\_t001 |  | Vitvi10g00390\_t001 |  |  |  |  |  |  |  |  |  |  |
| 1 | Vitvi19g00292\_t001 |  | \| \| |  |  |  |  |  |  |  |  |  |  |
| 1 | Vitvi19g04113\_t001 |  | \| \| |  |  |  |  |  |  |  |  |  |  |
| 1 | Vitvi19g04114\_t001 |  | Vitvi10g04259\_t002 |  |  |  |  |  |  |  |  |  |  |
| 1 | Vitvi19g01905\_t001 |  | \| \| |  |  |  |  |  |  |  |  |  |  |
| 1 | Vitvi19g01906\_t001 |  | \| \| |  |  |  |  |  |  |  |  |  |  |
| 1 | Vitvi19g04115\_t001 |  | \| \| |  |  |  |  |  |  |  |  |  |  |
| 1 | Vitvi19g04116\_t001 |  | \| \| |  |  |  |  |  |  |  |  |  |  |
| 1 | Vitvi19g01908\_t001 |  | \| \| |  |  |  |  |  |  |  |  |  |  |
| 1 | Vitvi19g00297\_t001 |  | \| \| |  |  |  |  |  |  |  |  |  |  |
| 1 | Vitvi19g00298\_t001 |  | Vitvi10g00372\_t001 |  |  |  |  |  |  |  |  |  |  |
| 0 | Vitvi19g00299\_t001 |  |  |  |  |  |  |  |  |  |  |  |  |
| 0 | Vitvi19g01910\_t001 |  |  |  |  |  |  |  |  |  |  |  |  |
| 0 | Vitvi19g00300\_t001 |  |  |  |  |  |  |  |  |  |  |  |  |
| 0 | Vitvi19g00302\_t001 |  |  |  |  |  |  |  |  |  |  |  |  |
| 0 | Vitvi19g00303\_t001 |  |  |  |  |  |  |  |  |  |  |  |  |
| 0 | Vitvi19g04117\_t001 |  |  |  |  |  |  |  |  |  |  |  |  |
| 0 | Vitvi19g00304\_t001 |  |  |  |  |  |  |  |  |  |  |  |  |
| 0 | Vitvi19g01912\_t001 |  |  |  |  |  |  |  |  |  |  |  |  |
| 0 | Vitvi19g01913\_t001 |  |  |  |  |  |  |  |  |  |  |  |  |
| 0 | Vitvi19g01914\_t001 |  |  |  |  |  |  |  |  |  |  |  |  |
| 0 | Vitvi19g00305\_t001 |  |  |  |  |  |  |  |  |  |  |  |  |
| 0 | Vitvi19g01916\_t001 |  |  |  |  |  |  |  |  |  |  |  |  |
| 0 | Vitvi19g04118\_t001 |  |  |  |  |  |  |  |  |  |  |  |  |
| 0 | Vitvi19g04119\_t001 |  |  |  |  |  |  |  |  |  |  |  |  |
| 0 | Vitvi19g00306\_t001 |  |  |  |  |  |  |  |  |  |  |  |  |
| 0 | Vitvi19g00307\_t001 |  |  |  |  |  |  |  |  |  |  |  |  |
| 0 | Vitvi19g00308\_t002 |  |  |  |  |  |  |  |  |  |  |  |  |
| 0 | Vitvi19g04120\_t001 |  |  |  |  |  |  |  |  |  |  |  |  |
| 0 | Vitvi19g04121\_t001 |  |  |  |  |  |  |  |  |  |  |  |  |
| 0 | Vitvi19g04122\_t001 |  |  |  |  |  |  |  |  |  |  |  |  |
| 0 | Vitvi19g04123\_t001 |  |  |  |  |  |  |  |  |  |  |  |  |
| 0 | Vitvi19g04124\_t001 |  |  |  |  |  |  |  |  |  |  |  |  |
| 0 | Vitvi19g01922\_t001 |  |  |  |  |  |  |  |  |  |  |  |  |
| 0 | Vitvi19g01923\_t001 |  |  |  |  |  |  |  |  |  |  |  |  |
| 0 | Vitvi19g01924\_t001 |  |  |  |  |  |  |  |  |  |  |  |  |
| 0 | Vitvi19g04125\_t001 |  |  |  |  |  |  |  |  |  |  |  |  |
| 0 | Vitvi19g04126\_t001 |  |  |  |  |  |  |  |  |  |  |  |  |
| 0 | Vitvi19g04127\_t001 |  |  |  |  |  |  |  |  |  |  |  |  |
| 0 | Vitvi19g04128\_t001 |  |  |  |  |  |  |  |  |  |  |  |  |
| 0 | Vitvi19g04129\_t001 |  |  |  |  |  |  |  |  |  |  |  |  |
| 0 | Vitvi19g04130\_t001 |  |  |  |  |  |  |  |  |  |  |  |  |
| 0 | Vitvi19g04131\_t001 |  |  |  |  |  |  |  |  |  |  |  |  |
| 0 | Vitvi19g04132\_t001 |  |  |  |  |  |  |  |  |  |  |  |  |
| 0 | Vitvi19g04133\_t001 |  |  |  |  |  |  |  |  |  |  |  |  |
| 0 | Vitvi19g04134\_t001 |  |  |  |  |  |  |  |  |  |  |  |  |
| 0 | Vitvi19g04135\_t001 |  |  |  |  |  |  |  |  |  |  |  |  |
| 0 | Vitvi19g00316\_t001 |  |  |  |  |  |  |  |  |  |  |  |  |
| 0 | Vitvi19g00317\_t001 |  |  |  |  |  |  |  |  |  |  |  |  |
| 0 | Vitvi19g00318\_t001 |  |  |  |  |  |  |  |  |  |  |  |  |
| 0 | Vitvi19g00319\_t001 |  |  |  |  |  |  |  |  |  |  |  |  |
| 0 | Vitvi19g00320\_t001 |  |  |  |  |  |  |  |  |  |  |  |  |
| 0 | Vitvi19g04136\_t001 |  |  |  |  |  |  |  |  |  |  |  |  |
| 0 | Vitvi19g04137\_t001 |  |  |  |  |  |  |  |  |  |  |  |  |
| 0 | Vitvi19g04138\_t001 |  |  |  |  |  |  |  |  |  |  |  |  |
| 0 | Vitvi19g00322\_t002 |  |  |  |  |  |  |  |  |  |  |  |  |
| 0 | Vitvi19g04139\_t001 |  |  |  |  |  |  |  |  |  |  |  |  |
| 0 | Vitvi19g01927\_t001 |  |  |  |  |  |  |  |  |  |  |  |  |
| 0 | Vitvi19g04140\_t001 |  |  |  |  |  |  |  |  |  |  |  |  |
| 0 | Vitvi19g04141\_t001 |  |  |  |  |  |  |  |  |  |  |  |  |
| 0 | Vitvi19g04142\_t001 |  |  |  |  |  |  |  |  |  |  |  |  |
| 0 | Vitvi19g01928\_t001 |  |  |  |  |  |  |  |  |  |  |  |  |
| 0 | Vitvi19g04143\_t001 |  |  |  |  |  |  |  |  |  |  |  |  |
| 0 | Vitvi19g02386\_t001 |  |  |  |  |  |  |  |  |  |  |  |  |
| 0 | Vitvi19g04144\_t001 |  |  |  |  |  |  |  |  |  |  |  |  |
| 0 | Vitvi19g04145\_t001 |  |  |  |  |  |  |  |  |  |  |  |  |
| 0 | Vitvi19g01929\_t001 |  |  |  |  |  |  |  |  |  |  |  |  |
| 0 | Vitvi19g01930\_t001 |  |  |  |  |  |  |  |  |  |  |  |  |
| 0 | Vitvi19g04146\_t001 |  |  |  |  |  |  |  |  |  |  |  |  |
| 0 | Vitvi19g01931\_t001 |  |  |  |  |  |  |  |  |  |  |  |  |
| 0 | Vitvi19g04147\_t001 |  |  |  |  |  |  |  |  |  |  |  |  |
| 0 | Vitvi19g04148\_t001 |  |  |  |  |  |  |  |  |  |  |  |  |
| 0 | Vitvi19g04149\_t001 |  |  |  |  |  |  |  |  |  |  |  |  |
| 0 | Vitvi19g04150\_t001 |  |  |  |  |  |  |  |  |  |  |  |  |
| 0 | Vitvi19g04151\_t001 |  |  |  |  |  |  |  |  |  |  |  |  |
| 0 | Vitvi19g00326\_t001 |  |  |  |  |  |  |  |  |  |  |  |  |
| 0 | Vitvi19g04152\_t001 |  |  |  |  |  |  |  |  |  |  |  |  |
| 0 | Vitvi19g04153\_t001 |  |  |  |  |  |  |  |  |  |  |  |  |
| 0 | Vitvi19g00328\_t001 |  |  |  |  |  |  |  |  |  |  |  |  |
| 0 | Vitvi19g00329\_t001 |  |  |  |  |  |  |  |  |  |  |  |  |
| 0 | Vitvi19g04154\_t001 |  |  |  |  |  |  |  |  |  |  |  |  |
| 0 | Vitvi19g04155\_t001 |  |  |  |  |  |  |  |  |  |  |  |  |
| 0 | Vitvi19g04156\_t001 |  |  |  |  |  |  |  |  |  |  |  |  |
| 0 | Vitvi19g04157\_t001 |  |  |  |  |  |  |  |  |  |  |  |  |
| 0 | Vitvi19g00331\_t001 |  |  |  |  |  |  |  |  |  |  |  |  |
| 0 | Vitvi19g00332\_t001 |  |  |  |  |  |  |  |  |  |  |  |  |
| 0 | Vitvi19g04158\_t001 |  |  |  |  |  |  |  |  |  |  |  |  |
| 0 | Vitvi19g04159\_t001 |  |  |  |  |  |  |  |  |  |  |  |  |
| 1 | Vitvi19g04160\_t001 |  | Vitvi12g00622\_t001 |  |  |  |  |  |  |  |  |  |  |
| 1 | Vitvi19g04161\_t001 |  | \| \| |  |  |  |  |  |  |  |  |  |  |
| 1 | Vitvi19g04162\_t001 |  | \| \| |  |  |  |  |  |  |  |  |  |  |
| 1 | Vitvi19g00339\_t001 |  | \| \| |  |  |  |  |  |  |  |  |  |  |
| 1 | Vitvi19g04163\_t001 |  | \| \| |  |  |  |  |  |  |  |  |  |  |
| 1 | Vitvi19g04164\_t001 |  | \| \| |  |  |  |  |  |  |  |  |  |  |
| 1 | Vitvi19g04165\_t001 |  | \| \| |  |  |  |  |  |  |  |  |  |  |
| 1 | Vitvi19g04166\_t001 |  | \| \| |  |  |  |  |  |  |  |  |  |  |
| 1 | Vitvi19g04167\_t001 |  | \| \| |  |  |  |  |  |  |  |  |  |  |
| 1 | Vitvi19g04168\_t001 |  | \| \| |  |  |  |  |  |  |  |  |  |  |
| 1 | Vitvi19g04169\_t001 |  | \| \| |  |  |  |  |  |  |  |  |  |  |
| 1 | Vitvi19g04170\_t001 |  | \| \| |  |  |  |  |  |  |  |  |  |  |
| 1 | Vitvi19g04171\_t001 |  | \| \| |  |  |  |  |  |  |  |  |  |  |
| 1 | Vitvi19g04172\_t001 |  | \| \| |  |  |  |  |  |  |  |  |  |  |
| 1 | Vitvi19g04173\_t001 |  | \| \| |  |  |  |  |  |  |  |  |  |  |
| 1 | Vitvi19g04174\_t001 |  | \| \| |  |  |  |  |  |  |  |  |  |  |
| 1 | Vitvi19g04175\_t001 |  | \| \| |  |  |  |  |  |  |  |  |  |  |
| 1 | Vitvi19g04176\_t001 |  | \| \| |  |  |  |  |  |  |  |  |  |  |
| 1 | Vitvi19g04177\_t001 |  | \| \| |  |  |  |  |  |  |  |  |  |  |
| 1 | Vitvi19g04178\_t001 |  | Vitvi12g02440\_t001 |  |  |  |  |  |  |  |  |  |  |
| 1 | Vitvi19g04179\_t001 |  | \| \| |  |  |  |  |  |  |  |  |  |  |
| 1 | Vitvi19g04180\_t001 |  | \| \| |  |  |  |  |  |  |  |  |  |  |
| 1 | Vitvi19g04181\_t001 |  | \| \| |  |  |  |  |  |  |  |  |  |  |
| 1 | Vitvi19g04182\_t001 |  | \| \| |  |  |  |  |  |  |  |  |  |  |
| 1 | Vitvi19g04183\_t001 |  | \| \| |  |  |  |  |  |  |  |  |  |  |
| 1 | Vitvi19g04184\_t001 |  | \| \| |  |  |  |  |  |  |  |  |  |  |
| 1 | Vitvi19g04185\_t001 |  | \| \| |  |  |  |  |  |  |  |  |  |  |
| 1 | Vitvi19g04186\_t001 |  | \| \| |  |  |  |  |  |  |  |  |  |  |
| 1 | Vitvi19g04187\_t001 |  | \| \| |  |  |  |  |  |  |  |  |  |  |
| 1 | Vitvi19g04188\_t001 |  | \| \| |  |  |  |  |  |  |  |  |  |  |
| 1 | Vitvi19g04189\_t001 |  | \| \| |  |  |  |  |  |  |  |  |  |  |
| 1 | Vitvi19g04190\_t001 |  | \| \| |  |  |  |  |  |  |  |  |  |  |
| 1 | Vitvi19g04191\_t001 |  | \| \| |  |  |  |  |  |  |  |  |  |  |
| 1 | Vitvi19g04192\_t001 |  | \| \| |  |  |  |  |  |  |  |  |  |  |
| 1 | Vitvi19g01952\_t003 |  | \| \| |  |  |  |  |  |  |  |  |  |  |
| 1 | Vitvi19g04193\_t001 |  | \| \| |  |  |  |  |  |  |  |  |  |  |
| 1 | Vitvi19g00364\_t001 |  | Vitvi12g00594\_t001 |  |  |  |  |  |  |  |  |  |  |
| 1 | Vitvi19g00366\_t001 |  | \| \| |  |  |  |  |  |  |  |  |  |  |
| 1 | Vitvi19g00367\_t001 |  | \| \| |  |  |  |  |  |  |  |  |  |  |
| 1 | Vitvi19g00368\_t001 |  | \| \| |  |  |  |  |  |  |  |  |  |  |
| 1 | Vitvi19g04194\_t001 |  | \| \| |  |  |  |  |  |  |  |  |  |  |
| 1 | Vitvi19g00369\_t001 |  | Vitvi12g00593\_t001 |  |  |  |  |  |  |  |  |  |  |
| 1 | Vitvi19g00370\_t003 |  | \| \| |  |  |  |  |  |  |  |  |  |  |
| 1 | Vitvi19g01972\_t001 |  | \| \| |  |  |  |  |  |  |  |  |  |  |
| 1 | Vitvi19g00371\_t001 |  | \| \| |  |  |  |  |  |  |  |  |  |  |
| 1 | Vitvi19g00372\_t001 |  | \| \| |  |  |  |  |  |  |  |  |  |  |
| 1 | Vitvi19g00373\_t001 |  | \| \| |  |  |  |  |  |  |  |  |  |  |
| 1 | Vitvi19g00375\_t001 |  | Vitvi12g00579\_t001 |  |  |  |  |  |  |  |  |  |  |
| 1 | Vitvi19g00376\_t001 |  | \| \| |  |  |  |  |  |  |  |  |  |  |
| 1 | Vitvi19g00377\_t001 |  | Vitvi12g02424\_t001 |  |  |  |  |  |  |  |  |  |  |
| 1 | Vitvi19g00378\_t002 |  | \| \| |  |  |  |  |  |  |  |  |  |  |
| 1 | Vitvi19g04195\_t001 |  | \| \| |  |  |  |  |  |  |  |  |  |  |
| 1 | Vitvi19g01791\_t003 |  | \| \| |  |  |  |  |  |  |  |  |  |  |
| 1 | Vitvi19g04196\_t001 |  | \| \| |  |  |  |  |  |  |  |  |  |  |
| 1 | Vitvi19g04197\_t001 |  | \| \| |  |  |  |  |  |  |  |  |  |  |
| 1 | Vitvi19g04198\_t001 |  | \| \| |  |  |  |  |  |  |  |  |  |  |
| 1 | Vitvi19g00384\_t001 |  | \| \| |  |  |  |  |  |  |  |  |  |  |
| 1 | Vitvi19g01974\_t001 |  | \| \| |  |  |  |  |  |  |  |  |  |  |
| 1 | Vitvi19g04199\_t001 |  | \| \| |  |  |  |  |  |  |  |  |  |  |
| 1 | Vitvi19g04200\_t001 |  | \| \| |  |  |  |  |  |  |  |  |  |  |
| 1 | Vitvi19g00387\_t001 |  | \| \| |  |  |  |  |  |  |  |  |  |  |
| 1 | Vitvi19g00388\_t001 |  | \| \| |  |  |  |  |  |  |  |  |  |  |
| 1 | Vitvi19g00389\_t001 |  | \| \| |  |  |  |  |  |  |  |  |  |  |
| 1 | Vitvi19g00391\_t001 |  | \| \| |  |  |  |  |  |  |  |  |  |  |
| 1 | Vitvi19g00392\_t001 |  | \| \| |  |  |  |  |  |  |  |  |  |  |
| 1 | Vitvi19g04201\_t001 |  | \| \| |  |  |  |  |  |  |  |  |  |  |
| 2 | Vitvi19g00394\_t001 |  | \| \| |  | Vitvi10g02192\_t001 |  |  |  |  |  |  |  |  |
| 2 | Vitvi19g00395\_t001 |  | \| \| |  | \| \| |  |  |  |  |  |  |  |  |
| 2 | Vitvi19g00396\_t001 |  | \| \| |  | \| \| |  |  |  |  |  |  |  |  |
| 2 | Vitvi19g00397\_t001 |  | \| \| |  | Vitvi10g00290\_t001 |  |  |  |  |  |  |  |  |
| 2 | Vitvi19g01977\_t001 |  | Vitvi12g00566\_t001 |  | Vitvi10g02202\_t001 |  |  |  |  |  |  |  |  |
| 2 | Vitvi19g00401\_t001 |  | \| \| |  | Vitvi10g02201\_t001 |  |  |  |  |  |  |  |  |
| 2 | Vitvi19g01979\_t001 |  | \| \| |  | \| \| |  |  |  |  |  |  |  |  |
| 2 | Vitvi19g00402\_t001 |  | Vitvi12g00560\_t001 |  | \| \| |  |  |  |  |  |  |  |  |
| 2 | Vitvi19g00403\_t001 |  | \| \| |  | \| \| |  |  |  |  |  |  |  |  |
| 2 | Vitvi19g00404\_t001 |  | \| \| |  | \| \| |  |  |  |  |  |  |  |  |
| 2 | Vitvi19g01981\_t001 |  | \| \| |  | Vitvi10g02212\_t001 |  |  |  |  |  |  |  |  |
| 2 | Vitvi19g00405\_t001 |  | \| \| |  | Vitvi10g02216\_t001 |  |  |  |  |  |  |  |  |
| 2 | Vitvi19g00406\_t001 |  | Vitvi12g00556\_t002 |  | Vitvi10g02218\_t001 |  |  |  |  |  |  |  |  |
| 1 | Vitvi19g00407\_t001 |  | \| \| |  |  |  |  |  |  |  |  |  |  |
| 1 | Vitvi19g00408\_t001 |  | Vitvi12g00542\_t001 |  |  |  |  |  |  |  |  |  |  |
| 0 | Vitvi19g00410\_t001 |  |  |  |  |  |  |  |  |  |  |  |  |
| 0 | Vitvi19g00411\_t001 |  |  |  |  |  |  |  |  |  |  |  |  |
| 0 | Vitvi19g04202\_t001 |  |  |  |  |  |  |  |  |  |  |  |  |
| 0 | Vitvi19g01982\_t001 |  |  |  |  |  |  |  |  |  |  |  |  |
| 0 | Vitvi19g00412\_t001 |  |  |  |  |  |  |  |  |  |  |  |  |
| 0 | Vitvi19g04203\_t001 |  |  |  |  |  |  |  |  |  |  |  |  |
| 0 | Vitvi19g00413\_t001 |  |  |  |  |  |  |  |  |  |  |  |  |
| 0 | Vitvi19g01983\_t001 |  |  |  |  |  |  |  |  |  |  |  |  |
| 0 | Vitvi19g04204\_t001 |  |  |  |  |  |  |  |  |  |  |  |  |
| 0 | Vitvi19g01985\_t003 |  |  |  |  |  |  |  |  |  |  |  |  |
| 0 | Vitvi19g01984\_t001 |  |  |  |  |  |  |  |  |  |  |  |  |
| 0 | Vitvi19g00416\_t001 |  |  |  |  |  |  |  |  |  |  |  |  |
| 0 | Vitvi19g00417\_t001 |  |  |  |  |  |  |  |  |  |  |  |  |
| 0 | Vitvi19g01986\_t001 |  |  |  |  |  |  |  |  |  |  |  |  |
| 0 | Vitvi19g00418\_t001 |  |  |  |  |  |  |  |  |  |  |  |  |
| 0 | Vitvi19g00420\_t002 |  |  |  |  |  |  |  |  |  |  |  |  |
| 0 | Vitvi19g04205\_t001 |  |  |  |  |  |  |  |  |  |  |  |  |
| 0 | Vitvi19g04206\_t001 |  |  |  |  |  |  |  |  |  |  |  |  |
| 0 | Vitvi19g04207\_t001 |  |  |  |  |  |  |  |  |  |  |  |  |
| 0 | Vitvi19g01987\_t001 |  |  |  |  |  |  |  |  |  |  |  |  |
| 0 | Vitvi19g04208\_t001 |  |  |  |  |  |  |  |  |  |  |  |  |
| 0 | Vitvi19g00421\_t001 |  |  |  |  |  |  |  |  |  |  |  |  |
| 0 | Vitvi19g04209\_t001 |  |  |  |  |  |  |  |  |  |  |  |  |
| 0 | Vitvi19g00423\_t001 |  |  |  |  |  |  |  |  |  |  |  |  |
| 0 | Vitvi19g04210\_t001 |  |  |  |  |  |  |  |  |  |  |  |  |
| 0 | Vitvi19g04211\_t001 |  |  |  |  |  |  |  |  |  |  |  |  |
| 0 | Vitvi19g00425\_t001 |  |  |  |  |  |  |  |  |  |  |  |  |
| 0 | Vitvi19g00426\_t001 |  |  |  |  |  |  |  |  |  |  |  |  |
| 0 | Vitvi19g04212\_t001 |  |  |  |  |  |  |  |  |  |  |  |  |
| 0 | Vitvi19g04213\_t001 |  |  |  |  |  |  |  |  |  |  |  |  |
| 0 | Vitvi19g04214\_t001 |  |  |  |  |  |  |  |  |  |  |  |  |
| 0 | Vitvi19g04215\_t001 |  |  |  |  |  |  |  |  |  |  |  |  |
| 0 | Vitvi19g01993\_t001 |  |  |  |  |  |  |  |  |  |  |  |  |
| 0 | Vitvi19g04216\_t001 |  |  |  |  |  |  |  |  |  |  |  |  |
| 0 | Vitvi19g00429\_t001 |  |  |  |  |  |  |  |  |  |  |  |  |
| 1 | Vitvi19g00431\_t001 |  | Vitvi10g00020\_t001 |  |  |  |  |  |  |  |  |  |  |
| 1 | Vitvi19g00432\_t001 |  | \| \| |  |  |  |  |  |  |  |  |  |  |
| 1 | Vitvi19g04217\_t001 |  | \| \| |  |  |  |  |  |  |  |  |  |  |
| 1 | Vitvi19g04218\_t001 |  | \| \| |  |  |  |  |  |  |  |  |  |  |
| 1 | Vitvi19g04219\_t001 |  | \| \| |  |  |  |  |  |  |  |  |  |  |
| 1 | Vitvi19g00434\_t001 |  | \| \| |  |  |  |  |  |  |  |  |  |  |
| 1 | Vitvi19g01995\_t001 |  | \| \| |  |  |  |  |  |  |  |  |  |  |
| 1 | Vitvi19g04220\_t001 |  | \| \| |  |  |  |  |  |  |  |  |  |  |
| 1 | Vitvi19g00435\_t001 |  | \| \| |  |  |  |  |  |  |  |  |  |  |
| 1 | Vitvi19g04221\_t001 |  | \| \| |  |  |  |  |  |  |  |  |  |  |
| 1 | Vitvi19g00436\_t001 |  | \| \| |  |  |  |  |  |  |  |  |  |  |
| 1 | Vitvi19g01997\_t001 |  | \| \| |  |  |  |  |  |  |  |  |  |  |
| 1 | Vitvi19g00441\_t001 |  | Vitvi10g00027\_t001 |  |  |  |  |  |  |  |  |  |  |
| 1 | Vitvi19g01998\_t002 |  | \| \| |  |  |  |  |  |  |  |  |  |  |
| 1 | Vitvi19g00442\_t001 |  | Vitvi10g00032\_t001 |  |  |  |  |  |  |  |  |  |  |
| 1 | Vitvi19g00443\_t001 |  | \| \| |  |  |  |  |  |  |  |  |  |  |
| 1 | Vitvi19g04222\_t001 |  | \| \| |  |  |  |  |  |  |  |  |  |  |
| 1 | Vitvi19g04223\_t001 |  | \| \| |  |  |  |  |  |  |  |  |  |  |
| 1 | Vitvi19g00445\_t001 |  | \| \| |  |  |  |  |  |  |  |  |  |  |
| 1 | Vitvi19g00446\_t001 |  | \| \| |  |  |  |  |  |  |  |  |  |  |
| 1 | Vitvi19g00447\_t001 |  | \| \| |  |  |  |  |  |  |  |  |  |  |
| 1 | Vitvi19g02000\_t001 |  | \| \| |  |  |  |  |  |  |  |  |  |  |
| 1 | Vitvi19g04224\_t001 |  | \| \| |  |  |  |  |  |  |  |  |  |  |
| 1 | Vitvi19g00449\_t001 |  | Vitvi10g00033\_t001 |  |  |  |  |  |  |  |  |  |  |
| 1 | Vitvi19g00451\_t001 |  | \| \| |  |  |  |  |  |  |  |  |  |  |
| 1 | Vitvi19g00453\_t001 |  | \| \| |  |  |  |  |  |  |  |  |  |  |
| 1 | Vitvi19g02001\_t001 |  | \| \| |  |  |  |  |  |  |  |  |  |  |
| 1 | Vitvi19g02002\_t001 |  | \| \| |  |  |  |  |  |  |  |  |  |  |
| 1 | Vitvi19g04225\_t001 |  | Vitvi10g01611\_t001 |  |  |  |  |  |  |  |  |  |  |
| 1 | Vitvi19g00456\_t001 |  | \| \| |  |  |  |  |  |  |  |  |  |  |
| 1 | Vitvi19g00457\_t001 |  | Vitvi10g00037\_t001 |  |  |  |  |  |  |  |  |  |  |
| 1 | Vitvi19g00458\_t001 |  | \| \| |  |  |  |  |  |  |  |  |  |  |
| 1 | Vitvi19g00459\_t001 |  | \| \| |  |  |  |  |  |  |  |  |  |  |
| 1 | Vitvi19g00460\_t001 |  | \| \| |  |  |  |  |  |  |  |  |  |  |
| 1 | Vitvi19g02003\_t001 |  | \| \| |  |  |  |  |  |  |  |  |  |  |
| 1 | Vitvi19g00462\_t001 |  | \| \| |  |  |  |  |  |  |  |  |  |  |
| 1 | Vitvi19g04226\_t001 |  | Vitvi10g01612\_t001 |  |  |  |  |  |  |  |  |  |  |
| 1 | Vitvi19g00463\_t001 |  | \| \| |  |  |  |  |  |  |  |  |  |  |
| 1 | Vitvi19g04227\_t001 |  | \| \| |  |  |  |  |  |  |  |  |  |  |
| 1 | Vitvi19g00464\_t001 |  | \| \| |  |  |  |  |  |  |  |  |  |  |
| 1 | Vitvi19g00465\_t001 |  | \| \| |  |  |  |  |  |  |  |  |  |  |
| 1 | Vitvi19g04228\_t001 |  | \| \| |  |  |  |  |  |  |  |  |  |  |
| 1 | Vitvi19g00467\_t001 |  | \| \| |  |  |  |  |  |  |  |  |  |  |
| 1 | Vitvi19g04229\_t001 |  | \| \| |  |  |  |  |  |  |  |  |  |  |
| 1 | Vitvi19g00469\_t001 |  | \| \| |  |  |  |  |  |  |  |  |  |  |
| 1 | Vitvi19g00470\_t001 |  | Vitvi10g00046\_t001 |  |  |  |  |  |  |  |  |  |  |
| 0 | Vitvi19g00471\_t001 |  |  |  |  |  |  |  |  |  |  |  |  |
| 0 | Vitvi19g00472\_t001 |  |  |  |  |  |  |  |  |  |  |  |  |
| 0 | Vitvi19g00473\_t001 |  |  |  |  |  |  |  |  |  |  |  |  |
| 0 | Vitvi19g00474\_t001 |  |  |  |  |  |  |  |  |  |  |  |  |
| 0 | Vitvi19g04230\_t001 |  |  |  |  |  |  |  |  |  |  |  |  |
| 0 | Vitvi19g04231\_t001 |  |  |  |  |  |  |  |  |  |  |  |  |
| 0 | Vitvi19g04232\_t001 |  |  |  |  |  |  |  |  |  |  |  |  |
| 0 | Vitvi19g00476\_t001 |  |  |  |  |  |  |  |  |  |  |  |  |
| 0 | Vitvi19g04233\_t001 |  |  |  |  |  |  |  |  |  |  |  |  |
| 0 | Vitvi19g02007\_t001 |  |  |  |  |  |  |  |  |  |  |  |  |
| 0 | Vitvi19g00477\_t001 |  |  |  |  |  |  |  |  |  |  |  |  |
| 0 | Vitvi19g04234\_t001 |  |  |  |  |  |  |  |  |  |  |  |  |
| 0 | Vitvi19g00478\_t001 |  |  |  |  |  |  |  |  |  |  |  |  |
| 0 | Vitvi19g02008\_t001 |  |  |  |  |  |  |  |  |  |  |  |  |
| 0 | Vitvi19g02009\_t001 |  |  |  |  |  |  |  |  |  |  |  |  |
| 0 | Vitvi19g00479\_t001 |  |  |  |  |  |  |  |  |  |  |  |  |
| 0 | Vitvi19g00480\_t001 |  |  |  |  |  |  |  |  |  |  |  |  |
| 0 | Vitvi19g00481\_t001 |  |  |  |  |  |  |  |  |  |  |  |  |
| 0 | Vitvi19g00482\_t001 |  |  |  |  |  |  |  |  |  |  |  |  |
| 0 | Vitvi19g00483\_t001 |  |  |  |  |  |  |  |  |  |  |  |  |
| 0 | Vitvi19g02010\_t001 |  |  |  |  |  |  |  |  |  |  |  |  |
| 0 | Vitvi19g00484\_t001 |  |  |  |  |  |  |  |  |  |  |  |  |
| 0 | Vitvi19g00486\_t001 |  |  |  |  |  |  |  |  |  |  |  |  |
| 0 | Vitvi19g00487\_t001 |  |  |  |  |  |  |  |  |  |  |  |  |
| 0 | Vitvi19g00488\_t001 |  |  |  |  |  |  |  |  |  |  |  |  |
| 0 | Vitvi19g00489\_t002 |  |  |  |  |  |  |  |  |  |  |  |  |
| 0 | Vitvi19g02011\_t001 |  |  |  |  |  |  |  |  |  |  |  |  |
| 0 | Vitvi19g04235\_t001 |  |  |  |  |  |  |  |  |  |  |  |  |
| 0 | Vitvi19g00490\_t001 |  |  |  |  |  |  |  |  |  |  |  |  |
| 0 | Vitvi19g00491\_t002 |  |  |  |  |  |  |  |  |  |  |  |  |
| 1 | Vitvi19g02012\_t001 |  | Vitvi10g01643\_t001 |  |  |  |  |  |  |  |  |  |  |
| 1 | Vitvi19g00492\_t001 |  | Vitvi10g00105\_t001 |  |  |  |  |  |  |  |  |  |  |
| 1 | Vitvi19g00493\_t001 |  | \| \| |  |  |  |  |  |  |  |  |  |  |
| 1 | Vitvi19g02013\_t001 |  | \| \| |  |  |  |  |  |  |  |  |  |  |
| 1 | Vitvi19g00494\_t001 |  | \| \| |  |  |  |  |  |  |  |  |  |  |
| 1 | Vitvi19g00497\_t001 |  | Vitvi10g00102\_t001 |  |  |  |  |  |  |  |  |  |  |
| 1 | Vitvi19g00498\_t001 |  | \| \| |  |  |  |  |  |  |  |  |  |  |
| 1 | Vitvi19g02014\_t001 |  | \| \| |  |  |  |  |  |  |  |  |  |  |
| 1 | Vitvi19g00499\_t001 |  | \| \| |  |  |  |  |  |  |  |  |  |  |
| 1 | Vitvi19g00501\_t001 |  | \| \| |  |  |  |  |  |  |  |  |  |  |
| 1 | Vitvi19g00502\_t001 |  | \| \| |  |  |  |  |  |  |  |  |  |  |
| 1 | Vitvi19g00503\_t001 |  | \| \| |  |  |  |  |  |  |  |  |  |  |
| 1 | Vitvi19g00504\_t001 |  | Vitvi10g00095\_t001 |  |  |  |  |  |  |  |  |  |  |
| 1 | Vitvi19g04236\_t001 |  | \| \| |  |  |  |  |  |  |  |  |  |  |
| 1 | Vitvi19g04237\_t001 |  | Vitvi10g00093\_t001 |  |  |  |  |  |  |  |  |  |  |
| 1 | Vitvi19g02017\_t001 |  | \| \| |  |  |  |  |  |  |  |  |  |  |
| 1 | Vitvi19g00506\_t001 |  | \| \| |  |  |  |  |  |  |  |  |  |  |
| 2 | Vitvi19g02020\_t001 |  | Vitvi10g00092\_t001 |  | Vitvi12g00369\_t001 |  |  |  |  |  |  |  |  |
| 2 | Vitvi19g00507\_t001 |  | \| \| |  | \| \| |  |  |  |  |  |  |  |  |
| 2 | Vitvi19g02021\_t001 |  | Vitvi10g04024\_t001 |  | Vitvi12g00370\_t002 |  |  |  |  |  |  |  |  |
| 2 | Vitvi19g00508\_t001 |  | \| \| |  | Vitvi12g00376\_t001 |  |  |  |  |  |  |  |  |
| 2 | Vitvi19g00509\_t001 |  | \| \| |  | \| \| |  |  |  |  |  |  |  |  |
| 2 | Vitvi19g04238\_t001 |  | \| \| |  | \| \| |  |  |  |  |  |  |  |  |
| 2 | Vitvi19g00512\_t001 |  | \| \| |  | \| \| |  |  |  |  |  |  |  |  |
| 2 | Vitvi19g04239\_t001 |  | \| \| |  | \| \| |  |  |  |  |  |  |  |  |
| 2 | Vitvi19g02023\_t001 |  | Vitvi10g00087\_t001 |  | \| \| |  |  |  |  |  |  |  |  |
| 2 | Vitvi19g00513\_t001 |  | \| \| |  | \| \| |  |  |  |  |  |  |  |  |
| 2 | Vitvi19g00514\_t001 |  | \| \| |  | \| \| |  |  |  |  |  |  |  |  |
| 2 | Vitvi19g00515\_t001 |  | \| \| |  | \| \| |  |  |  |  |  |  |  |  |
| 2 | Vitvi19g00518\_t001 |  | \| \| |  | \| \| |  |  |  |  |  |  |  |  |
| 2 | Vitvi19g00519\_t001 |  | \| \| |  | \| \| |  |  |  |  |  |  |  |  |
| 2 | Vitvi19g04240\_t001 |  | \| \| |  | \| \| |  |  |  |  |  |  |  |  |
| 2 | Vitvi19g04241\_t001 |  | \| \| |  | \| \| |  |  |  |  |  |  |  |  |
| 2 | Vitvi19g00521\_t001 |  | Vitvi10g00073\_t002 |  | \| \| |  |  |  |  |  |  |  |  |
| 2 | Vitvi19g00527\_t001 |  | \| \| |  | \| \| |  |  |  |  |  |  |  |  |
| 2 | Vitvi19g00528\_t001 |  | Vitvi10g01621\_t001 |  | Vitvi12g00387\_t001 |  |  |  |  |  |  |  |  |
| 2 | Vitvi19g00529\_t001 |  | Vitvi10g00064\_t001 |  | \| \| |  |  |  |  |  |  |  |  |
| 2 | Vitvi19g00530\_t001 |  | Vitvi10g00063\_t001 |  | Vitvi12g00388\_t001 |  |  |  |  |  |  |  |  |
| 2 | Vitvi19g04242\_t001 |  | \| \| |  | \| \| |  |  |  |  |  |  |  |  |
| 2 | Vitvi19g00533\_t001 |  | \| \| |  | \| \| |  |  |  |  |  |  |  |  |
| 2 | Vitvi19g00534\_t001 |  | Vitvi10g01619\_t001 |  | \| \| |  |  |  |  |  |  |  |  |
| 2 | Vitvi19g00535\_t001 |  | \| \| |  | \| \| |  |  |  |  |  |  |  |  |
| 2 | Vitvi19g02031\_t001 |  | Vitvi10g01616\_t001 |  | \| \| |  |  |  |  |  |  |  |  |
| 2 | Vitvi19g00536\_t001 |  | Vitvi10g01614\_t001 |  | \| \| |  |  |  |  |  |  |  |  |
| 2 | Vitvi19g00537\_t002 |  | Vitvi10g00057\_t001 |  | \| \| |  |  |  |  |  |  |  |  |
| 1 | Vitvi19g00538\_t001 |  |  |  | \| \| |  |  |  |  |  |  |  |  |
| 1 | Vitvi19g00539\_t002 |  |  |  | Vitvi12g00402\_t001 |  |  |  |  |  |  |  |  |
| 1 | Vitvi19g00541\_t001 |  |  |  | \| \| |  |  |  |  |  |  |  |  |
| 1 | Vitvi19g00542\_t001 |  |  |  | \| \| |  |  |  |  |  |  |  |  |
| 1 | Vitvi19g02032\_t001 |  |  |  | \| \| |  |  |  |  |  |  |  |  |
| 1 | Vitvi19g00543\_t001 |  |  |  | Vitvi12g00406\_t001 |  |  |  |  |  |  |  |  |
| 1 | Vitvi19g00547\_t002 |  |  |  | Vitvi12g00409\_t001 |  |  |  |  |  |  |  |  |
| 1 | Vitvi19g00549\_t001 |  |  |  | \| \| |  |  |  |  |  |  |  |  |
| 1 | Vitvi19g00550\_t001 |  |  |  | \| \| |  |  |  |  |  |  |  |  |
| 1 | Vitvi19g00551\_t001 |  |  |  | \| \| |  |  |  |  |  |  |  |  |
| 1 | Vitvi19g00552\_t001 |  |  |  | \| \| |  |  |  |  |  |  |  |  |
| 1 | Vitvi19g00554\_t001 |  |  |  | \| \| |  |  |  |  |  |  |  |  |
| 1 | Vitvi19g00555\_t001 |  |  |  | Vitvi12g00430\_t001 |  |  |  |  |  |  |  |  |
| 1 | Vitvi19g00557\_t001 |  |  |  | Vitvi12g00440\_t001 |  |  |  |  |  |  |  |  |
| 1 | Vitvi19g00558\_t001 |  |  |  | Vitvi12g00451\_t001 |  |  |  |  |  |  |  |  |
| 1 | Vitvi19g04243\_t001 |  |  |  | \| \| |  |  |  |  |  |  |  |  |
| 1 | Vitvi19g00559\_t001 |  |  |  | \| \| |  |  |  |  |  |  |  |  |
| 2 | Vitvi19g00561\_t001 |  | Vitvi10g00164\_t001 |  | \| \| |  |  |  |  |  |  |  |  |
| 2 | Vitvi19g04244\_t001 |  | \| \| |  | \| \| |  |  |  |  |  |  |  |  |
| 2 | Vitvi19g04245\_t001 |  | \| \| |  | \| \| |  |  |  |  |  |  |  |  |
| 2 | Vitvi19g00563\_t001 |  | \| \| |  | \| \| |  |  |  |  |  |  |  |  |
| 2 | Vitvi19g00564\_t001 |  | \| \| |  | Vitvi12g04138\_t001 |  |  |  |  |  |  |  |  |
| 2 | Vitvi19g00565\_t001 |  | \| \| |  | Vitvi12g02387\_t001 |  |  |  |  |  |  |  |  |
| 2 | Vitvi19g00566\_t001 |  | \| \| |  | \| \| |  |  |  |  |  |  |  |  |
| 2 | Vitvi19g00567\_t001 |  | \| \| |  | \| \| |  |  |  |  |  |  |  |  |
| 2 | Vitvi19g00568\_t001 |  | Vitvi10g04073\_t001 |  | \| \| |  |  |  |  |  |  |  |  |
| 2 | Vitvi19g00569\_t001 |  | \| \| |  | \| \| |  |  |  |  |  |  |  |  |
| 2 | Vitvi19g00570\_t001 |  | Vitvi10g00174\_t002 |  | \| \| |  |  |  |  |  |  |  |  |
| 2 | Vitvi19g00572\_t001 |  | \| \| |  | \| \| |  |  |  |  |  |  |  |  |
| 2 | Vitvi19g00573\_t001 |  | \| \| |  | \| \| |  |  |  |  |  |  |  |  |
| 2 | Vitvi19g00574\_t001 |  | \| \| |  | \| \| |  |  |  |  |  |  |  |  |
| 2 | Vitvi19g00575\_t001 |  | \| \| |  | \| \| |  |  |  |  |  |  |  |  |
| 2 | Vitvi19g00576\_t001 |  | \| \| |  | Vitvi12g00466\_t001 |  |  |  |  |  |  |  |  |
| 1 | Vitvi19g00577\_t003 |  | \| \| |  |  |  |  |  |  |  |  |  |  |
| 1 | Vitvi19g02035\_t001 |  | \| \| |  |  |  |  |  |  |  |  |  |  |
| 1 | Vitvi19g00578\_t002 |  | \| \| |  |  |  |  |  |  |  |  |  |  |
| 1 | Vitvi19g00579\_t001 |  | \| \| |  |  |  |  |  |  |  |  |  |  |
| 1 | Vitvi19g00580\_t001 |  | Vitvi10g01667\_t001 |  |  |  |  |  |  |  |  |  |  |
| 1 | Vitvi19g00581\_t001 |  | \| \| |  |  |  |  |  |  |  |  |  |  |
| 1 | Vitvi19g04246\_t001 |  | \| \| |  |  |  |  |  |  |  |  |  |  |
| 1 | Vitvi19g04247\_t001 |  | \| \| |  |  |  |  |  |  |  |  |  |  |
| 1 | Vitvi19g02038\_t001 |  | \| \| |  |  |  |  |  |  |  |  |  |  |
| 1 | Vitvi19g02039\_t001 |  | \| \| |  |  |  |  |  |  |  |  |  |  |
| 1 | Vitvi19g02040\_t001 |  | \| \| |  |  |  |  |  |  |  |  |  |  |
| 1 | Vitvi19g04248\_t001 |  | \| \| |  |  |  |  |  |  |  |  |  |  |
| 1 | Vitvi19g00585\_t001 |  | Vitvi10g00183\_t001 |  |  |  |  |  |  |  |  |  |  |
| 1 | Vitvi19g00586\_t001 |  | Vitvi10g04078\_t001 |  |  |  |  |  |  |  |  |  |  |
| 1 | Vitvi19g00587\_t001 |  | \| \| |  |  |  |  |  |  |  |  |  |  |
| 1 | Vitvi19g02042\_t001 |  | \| \| |  |  |  |  |  |  |  |  |  |  |
| 1 | Vitvi19g00588\_t001 |  | \| \| |  |  |  |  |  |  |  |  |  |  |
| 1 | Vitvi19g00589\_t001 |  | \| \| |  |  |  |  |  |  |  |  |  |  |
| 1 | Vitvi19g00590\_t001 |  | Vitvi10g00191\_t001 |  |  |  |  |  |  |  |  |  |  |
| 1 | Vitvi19g00591\_t001 |  | Vitvi10g04080\_t001 |  |  |  |  |  |  |  |  |  |  |
| 0 | Vitvi19g00592\_t001 |  |  |  |  |  |  |  |  |  |  |  |  |
| 0 | Vitvi19g04249\_t001 |  |  |  |  |  |  |  |  |  |  |  |  |
| 0 | Vitvi19g00596\_t001 |  |  |  |  |  |  |  |  |  |  |  |  |
| 0 | Vitvi19g04250\_t001 |  |  |  |  |  |  |  |  |  |  |  |  |
| 0 | Vitvi19g04251\_t001 |  |  |  |  |  |  |  |  |  |  |  |  |
| 1 | Vitvi19g00600\_t001 |  | Vitvi12g00645\_t001 |  |  |  |  |  |  |  |  |  |  |
| 1 | Vitvi19g00601\_t001 |  | \| \| |  |  |  |  |  |  |  |  |  |  |
| 1 | Vitvi19g00602\_t001 |  | \| \| |  |  |  |  |  |  |  |  |  |  |
| 1 | Vitvi19g00604\_t001 |  | Vitvi12g00648\_t001 |  |  |  |  |  |  |  |  |  |  |
| 1 | Vitvi19g04252\_t001 |  | \| \| |  |  |  |  |  |  |  |  |  |  |
| 1 | Vitvi19g00606\_t001 |  | \| \| |  |  |  |  |  |  |  |  |  |  |
| 1 | Vitvi19g00607\_t001 |  | \| \| |  |  |  |  |  |  |  |  |  |  |
| 1 | Vitvi19g04253\_t001 |  | \| \| |  |  |  |  |  |  |  |  |  |  |
| 1 | Vitvi19g00608\_t001 |  | \| \| |  |  |  |  |  |  |  |  |  |  |
| 1 | Vitvi19g00609\_t001 |  | \| \| |  |  |  |  |  |  |  |  |  |  |
| 1 | Vitvi19g00610\_t001 |  | Vitvi12g00655\_t001 |  |  |  |  |  |  |  |  |  |  |
| 1 | Vitvi19g04254\_t001 |  | \| \| |  |  |  |  |  |  |  |  |  |  |
| 1 | Vitvi19g02047\_t001 |  | \| \| |  |  |  |  |  |  |  |  |  |  |
| 1 | Vitvi19g00611\_t001 |  | \| \| |  |  |  |  |  |  |  |  |  |  |
| 1 | Vitvi19g00612\_t001 |  | Vitvi12g00656\_t001 |  |  |  |  |  |  |  |  |  |  |
| 1 | Vitvi19g00613\_t001 |  | \| \| |  |  |  |  |  |  |  |  |  |  |
| 1 | Vitvi19g00614\_t001 |  | Vitvi12g04217\_t001 |  |  |  |  |  |  |  |  |  |  |
| 1 | Vitvi19g00615\_t001 |  | \| \| |  |  |  |  |  |  |  |  |  |  |
| 1 | Vitvi19g00617\_t001 |  | Vitvi12g00664\_t003 |  |  |  |  |  |  |  |  |  |  |
| 1 | Vitvi19g00618\_t001 |  | \| \| |  |  |  |  |  |  |  |  |  |  |
| 1 | Vitvi19g00619\_t001 |  | Vitvi12g00665\_t002 |  |  |  |  |  |  |  |  |  |  |
| 1 | Vitvi19g04255\_t001 |  | \| \| |  |  |  |  |  |  |  |  |  |  |
| 1 | Vitvi19g00620\_t001 |  | \| \| |  |  |  |  |  |  |  |  |  |  |
| 1 | Vitvi19g00621\_t001 |  | \| \| |  |  |  |  |  |  |  |  |  |  |
| 1 | Vitvi19g04256\_t001 |  | \| \| |  |  |  |  |  |  |  |  |  |  |
| 1 | Vitvi19g00623\_t003 |  | Vitvi12g00667\_t001 |  |  |  |  |  |  |  |  |  |  |
| 1 | Vitvi19g00625\_t001 |  | \| \| |  |  |  |  |  |  |  |  |  |  |
| 1 | Vitvi19g00626\_t001 |  | \| \| |  |  |  |  |  |  |  |  |  |  |
| 1 | Vitvi19g04257\_t001 |  | Vitvi12g02486\_t001 |  |  |  |  |  |  |  |  |  |  |
| 0 | Vitvi19g00627\_t001 |  |  |  |  |  |  |  |  |  |  |  |  |
| 0 | Vitvi19g00630\_t001 |  |  |  |  |  |  |  |  |  |  |  |  |
| 0 | Vitvi19g04258\_t001 |  |  |  |  |  |  |  |  |  |  |  |  |
| 0 | Vitvi19g00631\_t001 |  |  |  |  |  |  |  |  |  |  |  |  |
| 0 | Vitvi19g04259\_t001 |  |  |  |  |  |  |  |  |  |  |  |  |
| 0 | Vitvi19g00633\_t001 |  |  |  |  |  |  |  |  |  |  |  |  |
| 0 | Vitvi19g04260\_t001 |  |  |  |  |  |  |  |  |  |  |  |  |
| 0 | Vitvi19g04261\_t001 |  |  |  |  |  |  |  |  |  |  |  |  |
| 0 | Vitvi19g00640\_t001 |  |  |  |  |  |  |  |  |  |  |  |  |
| 0 | Vitvi19g00641\_t002 |  |  |  |  |  |  |  |  |  |  |  |  |
| 0 | Vitvi19g00645\_t001 |  |  |  |  |  |  |  |  |  |  |  |  |
| 0 | Vitvi19g04262\_t001 |  |  |  |  |  |  |  |  |  |  |  |  |
| 0 | Vitvi19g04263\_t001 |  |  |  |  |  |  |  |  |  |  |  |  |
| 0 | Vitvi19g04264\_t001 |  |  |  |  |  |  |  |  |  |  |  |  |
| 0 | Vitvi19g00652\_t001 |  |  |  |  |  |  |  |  |  |  |  |  |
| 0 | Vitvi19g00653\_t001 |  |  |  |  |  |  |  |  |  |  |  |  |
| 0 | Vitvi19g00654\_t001 |  |  |  |  |  |  |  |  |  |  |  |  |
| 0 | Vitvi19g04265\_t001 |  |  |  |  |  |  |  |  |  |  |  |  |
| 0 | Vitvi19g00655\_t001 |  |  |  |  |  |  |  |  |  |  |  |  |
| 0 | Vitvi19g00656\_t001 |  |  |  |  |  |  |  |  |  |  |  |  |
| 0 | Vitvi19g00657\_t002 |  |  |  |  |  |  |  |  |  |  |  |  |
| 0 | Vitvi19g00658\_t001 |  |  |  |  |  |  |  |  |  |  |  |  |
| 0 | Vitvi19g00659\_t001 |  |  |  |  |  |  |  |  |  |  |  |  |
| 0 | Vitvi19g02052\_t001 |  |  |  |  |  |  |  |  |  |  |  |  |
| 0 | Vitvi19g02056\_t001 |  |  |  |  |  |  |  |  |  |  |  |  |
| 0 | Vitvi19g04266\_t001 |  |  |  |  |  |  |  |  |  |  |  |  |
| 0 | Vitvi19g02057\_t001 |  |  |  |  |  |  |  |  |  |  |  |  |
| 1 | Vitvi19g00660\_t001 |  | Vitvi12g00706\_t001 |  |  |  |  |  |  |  |  |  |  |
| 1 | Vitvi19g02058\_t001 |  | Vitvi12g02493\_t001 |  |  |  |  |  |  |  |  |  |  |
| 1 | Vitvi19g00662\_t001 |  | \| \| |  |  |  |  |  |  |  |  |  |  |
| 1 | Vitvi19g02059\_t001 |  | \| \| |  |  |  |  |  |  |  |  |  |  |
| 1 | Vitvi19g02060\_t001 |  | \| \| |  |  |  |  |  |  |  |  |  |  |
| 1 | Vitvi19g00664\_t001 |  | \| \| |  |  |  |  |  |  |  |  |  |  |
| 1 | Vitvi19g02061\_t001 |  | \| \| |  |  |  |  |  |  |  |  |  |  |
| 1 | Vitvi19g04267\_t001 |  | \| \| |  |  |  |  |  |  |  |  |  |  |
| 1 | Vitvi19g00665\_t001 |  | \| \| |  |  |  |  |  |  |  |  |  |  |
| 1 | Vitvi19g00666\_t001 |  | \| \| |  |  |  |  |  |  |  |  |  |  |
| 1 | Vitvi19g00668\_t001 |  | \| \| |  |  |  |  |  |  |  |  |  |  |
| 1 | Vitvi19g02062\_t001 |  | Vitvi12g02494\_t001 |  |  |  |  |  |  |  |  |  |  |
| 1 | Vitvi19g04268\_t001 |  | \| \| |  |  |  |  |  |  |  |  |  |  |
| 1 | Vitvi19g04269\_t001 |  | \| \| |  |  |  |  |  |  |  |  |  |  |
| 1 | Vitvi19g04270\_t001 |  | \| \| |  |  |  |  |  |  |  |  |  |  |
| 1 | Vitvi19g04271\_t001 |  | \| \| |  |  |  |  |  |  |  |  |  |  |
| 1 | Vitvi19g04272\_t001 |  | \| \| |  |  |  |  |  |  |  |  |  |  |
| 1 | Vitvi19g00672\_t001 |  | \| \| |  |  |  |  |  |  |  |  |  |  |
| 1 | Vitvi19g04273\_t001 |  | \| \| |  |  |  |  |  |  |  |  |  |  |
| 1 | Vitvi19g00673\_t001 |  | Vitvi12g00719\_t001 |  |  |  |  |  |  |  |  |  |  |
| 1 | Vitvi19g00674\_t001 |  | \| \| |  |  |  |  |  |  |  |  |  |  |
| 1 | Vitvi19g00675\_t001 |  | \| \| |  |  |  |  |  |  |  |  |  |  |
| 1 | Vitvi19g00676\_t001 |  | \| \| |  |  |  |  |  |  |  |  |  |  |
| 1 | Vitvi19g00677\_t001 |  | \| \| |  |  |  |  |  |  |  |  |  |  |
| 1 | Vitvi19g04274\_t001 |  | \| \| |  |  |  |  |  |  |  |  |  |  |
| 1 | Vitvi19g00678\_t001 |  | \| \| |  |  |  |  |  |  |  |  |  |  |
| 1 | Vitvi19g00680\_t001 |  | \| \| |  |  |  |  |  |  |  |  |  |  |
| 1 | Vitvi19g00681\_t001 |  | \| \| |  |  |  |  |  |  |  |  |  |  |
| 1 | Vitvi19g00682\_t001 |  | Vitvi12g00721\_t001 |  |  |  |  |  |  |  |  |  |  |
| 1 | Vitvi19g02065\_t001 |  | \| \| |  |  |  |  |  |  |  |  |  |  |
| 1 | Vitvi19g00683\_t001 |  | Vitvi12g04246\_t001 |  |  |  |  |  |  |  |  |  |  |
| 1 | Vitvi19g00685\_t001 |  | \| \| |  |  |  |  |  |  |  |  |  |  |
| 1 | Vitvi19g00686\_t001 |  | \| \| |  |  |  |  |  |  |  |  |  |  |
| 1 | Vitvi19g02067\_t001 |  | \| \| |  |  |  |  |  |  |  |  |  |  |
| 1 | Vitvi19g00687\_t007 |  | \| \| |  |  |  |  |  |  |  |  |  |  |
| 1 | Vitvi19g00688\_t001 |  | \| \| |  |  |  |  |  |  |  |  |  |  |
| 1 | Vitvi19g00690\_t001 |  | \| \| |  |  |  |  |  |  |  |  |  |  |
| 1 | Vitvi19g00691\_t001 |  | \| \| |  |  |  |  |  |  |  |  |  |  |
| 1 | Vitvi19g00692\_t001 |  | \| \| |  |  |  |  |  |  |  |  |  |  |
| 1 | Vitvi19g00693\_t001 |  | \| \| |  |  |  |  |  |  |  |  |  |  |
| 1 | Vitvi19g02068\_t001 |  | \| \| |  |  |  |  |  |  |  |  |  |  |
| 1 | Vitvi19g00694\_t002 |  | \| \| |  |  |  |  |  |  |  |  |  |  |
| 1 | Vitvi19g00695\_t001 |  | Vitvi12g04252\_t001 |  |  |  |  |  |  |  |  |  |  |
| 1 | Vitvi19g00696\_t001 |  | Vitvi12g00741\_t001 |  |  |  |  |  |  |  |  |  |  |
| 0 | Vitvi19g00697\_t001 |  |  |  |  |  |  |  |  |  |  |  |  |
| 0 | Vitvi19g00698\_t001 |  |  |  |  |  |  |  |  |  |  |  |  |
| 0 | Vitvi19g00700\_t001 |  |  |  |  |  |  |  |  |  |  |  |  |
| 0 | Vitvi19g04275\_t001 |  |  |  |  |  |  |  |  |  |  |  |  |
| 0 | Vitvi19g04276\_t001 |  |  |  |  |  |  |  |  |  |  |  |  |
| 0 | Vitvi19g00703\_t001 |  |  |  |  |  |  |  |  |  |  |  |  |
| 0 | Vitvi19g00704\_t001 |  |  |  |  |  |  |  |  |  |  |  |  |
| 0 | Vitvi19g00706\_t001 |  |  |  |  |  |  |  |  |  |  |  |  |
| 0 | Vitvi19g00707\_t001 |  |  |  |  |  |  |  |  |  |  |  |  |
| 0 | Vitvi19g04277\_t001 |  |  |  |  |  |  |  |  |  |  |  |  |
| 0 | Vitvi19g00708\_t001 |  |  |  |  |  |  |  |  |  |  |  |  |
| 0 | Vitvi19g04278\_t001 |  |  |  |  |  |  |  |  |  |  |  |  |
| 0 | Vitvi19g04279\_t001 |  |  |  |  |  |  |  |  |  |  |  |  |
| 0 | Vitvi19g04280\_t001 |  |  |  |  |  |  |  |  |  |  |  |  |
| 0 | Vitvi19g00709\_t001 |  |  |  |  |  |  |  |  |  |  |  |  |
| 0 | Vitvi19g04281\_t003 |  |  |  |  |  |  |  |  |  |  |  |  |
| 0 | Vitvi19g04282\_t001 |  |  |  |  |  |  |  |  |  |  |  |  |
| 0 | Vitvi19g00712\_t001 |  |  |  |  |  |  |  |  |  |  |  |  |
| 0 | Vitvi19g00713\_t001 |  |  |  |  |  |  |  |  |  |  |  |  |
| 0 | Vitvi19g00714\_t001 |  |  |  |  |  |  |  |  |  |  |  |  |
| 0 | Vitvi19g04283\_t001 |  |  |  |  |  |  |  |  |  |  |  |  |
| 0 | Vitvi19g02073\_t001 |  |  |  |  |  |  |  |  |  |  |  |  |
| 0 | Vitvi19g04284\_t001 |  |  |  |  |  |  |  |  |  |  |  |  |
| 0 | Vitvi19g00719\_t001 |  |  |  |  |  |  |  |  |  |  |  |  |
| 0 | Vitvi19g04285\_t001 |  |  |  |  |  |  |  |  |  |  |  |  |
| 0 | Vitvi19g00720\_t001 |  |  |  |  |  |  |  |  |  |  |  |  |
| 0 | Vitvi19g00721\_t001 |  |  |  |  |  |  |  |  |  |  |  |  |
| 0 | Vitvi19g00722\_t001 |  |  |  |  |  |  |  |  |  |  |  |  |
| 0 | Vitvi19g00723\_t001 |  |  |  |  |  |  |  |  |  |  |  |  |
| 0 | Vitvi19g00724\_t001 |  |  |  |  |  |  |  |  |  |  |  |  |
| 0 | Vitvi19g00725\_t001 |  |  |  |  |  |  |  |  |  |  |  |  |
| 0 | Vitvi19g00726\_t001 |  |  |  |  |  |  |  |  |  |  |  |  |
| 0 | Vitvi19g04286\_t001 |  |  |  |  |  |  |  |  |  |  |  |  |
| 0 | Vitvi19g04287\_t001 |  |  |  |  |  |  |  |  |  |  |  |  |
| 0 | Vitvi19g00727\_t007 |  |  |  |  |  |  |  |  |  |  |  |  |
| 0 | Vitvi19g00728\_t001 |  |  |  |  |  |  |  |  |  |  |  |  |
| 0 | Vitvi19g00729\_t001 |  |  |  |  |  |  |  |  |  |  |  |  |
| 0 | Vitvi19g02076\_t001 |  |  |  |  |  |  |  |  |  |  |  |  |
| 0 | Vitvi19g04288\_t001 |  |  |  |  |  |  |  |  |  |  |  |  |
| 0 | Vitvi19g02077\_t001 |  |  |  |  |  |  |  |  |  |  |  |  |
| 0 | Vitvi19g04289\_t001 |  |  |  |  |  |  |  |  |  |  |  |  |
| 0 | Vitvi19g00730\_t001 |  |  |  |  |  |  |  |  |  |  |  |  |
| 0 | Vitvi19g00731\_t001 |  |  |  |  |  |  |  |  |  |  |  |  |
| 0 | Vitvi19g02080\_t001 |  |  |  |  |  |  |  |  |  |  |  |  |
| 0 | Vitvi19g00732\_t001 |  |  |  |  |  |  |  |  |  |  |  |  |
| 0 | Vitvi19g00734\_t001 |  |  |  |  |  |  |  |  |  |  |  |  |
| 0 | Vitvi19g00735\_t001 |  |  |  |  |  |  |  |  |  |  |  |  |
| 0 | Vitvi19g00736\_t001 |  |  |  |  |  |  |  |  |  |  |  |  |
| 0 | Vitvi19g00738\_t001 |  |  |  |  |  |  |  |  |  |  |  |  |
| 0 | Vitvi19g00739\_t001 |  |  |  |  |  |  |  |  |  |  |  |  |
| 0 | Vitvi19g00740\_t001 |  |  |  |  |  |  |  |  |  |  |  |  |
| 0 | Vitvi19g02081\_t001 |  |  |  |  |  |  |  |  |  |  |  |  |
| 0 | Vitvi19g00741\_t001 |  |  |  |  |  |  |  |  |  |  |  |  |
| 0 | Vitvi19g00743\_t001 |  |  |  |  |  |  |  |  |  |  |  |  |
| 0 | Vitvi19g00744\_t002 |  |  |  |  |  |  |  |  |  |  |  |  |
| 0 | Vitvi19g04290\_t001 |  |  |  |  |  |  |  |  |  |  |  |  |
| 0 | Vitvi19g00745\_t001 |  |  |  |  |  |  |  |  |  |  |  |  |
| 0 | Vitvi19g00747\_t001 |  |  |  |  |  |  |  |  |  |  |  |  |
| 0 | Vitvi19g00749\_t001 |  |  |  |  |  |  |  |  |  |  |  |  |
| 0 | Vitvi19g00750\_t001.1.6037826d |  |  |  |  |  |  |  |  |  |  |  |  |
| 0 | Vitvi19g02084\_t002 |  |  |  |  |  |  |  |  |  |  |  |  |
| 0 | Vitvi19g00752\_t001 |  |  |  |  |  |  |  |  |  |  |  |  |
| 0 | Vitvi19g00757\_t001 |  |  |  |  |  |  |  |  |  |  |  |  |
| 0 | Vitvi19g00758\_t001 |  |  |  |  |  |  |  |  |  |  |  |  |
| 0 | Vitvi19g00759\_t001 |  |  |  |  |  |  |  |  |  |  |  |  |
| 0 | Vitvi19g00760\_t001 |  |  |  |  |  |  |  |  |  |  |  |  |
| 0 | Vitvi19g00761\_t001 |  |  |  |  |  |  |  |  |  |  |  |  |
| 0 | Vitvi19g00762\_t002 |  |  |  |  |  |  |  |  |  |  |  |  |
| 0 | Vitvi19g00766\_t001 |  |  |  |  |  |  |  |  |  |  |  |  |
| 0 | Vitvi19g00767\_t001 |  |  |  |  |  |  |  |  |  |  |  |  |
| 0 | Vitvi19g00768\_t001 |  |  |  |  |  |  |  |  |  |  |  |  |
| 0 | Vitvi19g04291\_t001 |  |  |  |  |  |  |  |  |  |  |  |  |
| 0 | Vitvi19g04292\_t001 |  |  |  |  |  |  |  |  |  |  |  |  |
| 0 | Vitvi19g00775\_t001 |  |  |  |  |  |  |  |  |  |  |  |  |
| 0 | Vitvi19g00776\_t001 |  |  |  |  |  |  |  |  |  |  |  |  |
| 0 | Vitvi19g04293\_t001 |  |  |  |  |  |  |  |  |  |  |  |  |
| 0 | Vitvi19g00778\_t001 |  |  |  |  |  |  |  |  |  |  |  |  |
| 0 | Vitvi19g04294\_t001 |  |  |  |  |  |  |  |  |  |  |  |  |
| 0 | Vitvi19g04295\_t001 |  |  |  |  |  |  |  |  |  |  |  |  |
| 0 | Vitvi19g00780\_t001 |  |  |  |  |  |  |  |  |  |  |  |  |
| 0 | Vitvi19g00781\_t001 |  |  |  |  |  |  |  |  |  |  |  |  |
| 0 | Vitvi19g00782\_t001 |  |  |  |  |  |  |  |  |  |  |  |  |
| 0 | Vitvi19g00783\_t001 |  |  |  |  |  |  |  |  |  |  |  |  |
| 0 | Vitvi19g04296\_t001 |  |  |  |  |  |  |  |  |  |  |  |  |
| 0 | Vitvi19g00786\_t001 |  |  |  |  |  |  |  |  |  |  |  |  |
| 0 | Vitvi19g00787\_t001 |  |  |  |  |  |  |  |  |  |  |  |  |
| 0 | Vitvi19g02088\_t001 |  |  |  |  |  |  |  |  |  |  |  |  |
| 0 | Vitvi19g02091\_t001 |  |  |  |  |  |  |  |  |  |  |  |  |
| 0 | Vitvi19g00788\_t001 |  |  |  |  |  |  |  |  |  |  |  |  |
| 0 | Vitvi19g00789\_t001 |  |  |  |  |  |  |  |  |  |  |  |  |
| 0 | Vitvi19g02092\_t001 |  |  |  |  |  |  |  |  |  |  |  |  |
| 0 | Vitvi19g04297\_t001 |  |  |  |  |  |  |  |  |  |  |  |  |
| 0 | Vitvi19g00791\_t001 |  |  |  |  |  |  |  |  |  |  |  |  |
| 0 | Vitvi19g04298\_t001 |  |  |  |  |  |  |  |  |  |  |  |  |
| 0 | Vitvi19g02097\_t001 |  |  |  |  |  |  |  |  |  |  |  |  |
| 0 | Vitvi19g02099\_t001 |  |  |  |  |  |  |  |  |  |  |  |  |
| 0 | Vitvi19g02100\_t001 |  |  |  |  |  |  |  |  |  |  |  |  |
| 0 | Vitvi19g04299\_t001 |  |  |  |  |  |  |  |  |  |  |  |  |
| 0 | Vitvi19g00794\_t001 |  |  |  |  |  |  |  |  |  |  |  |  |
| 0 | Vitvi19g00795\_t001 |  |  |  |  |  |  |  |  |  |  |  |  |
| 0 | Vitvi19g02101\_t001 |  |  |  |  |  |  |  |  |  |  |  |  |
| 0 | Vitvi19g02102\_t001 |  |  |  |  |  |  |  |  |  |  |  |  |
| 0 | Vitvi19g04300\_t001 |  |  |  |  |  |  |  |  |  |  |  |  |
| 0 | Vitvi19g04301\_t001 |  |  |  |  |  |  |  |  |  |  |  |  |
| 0 | Vitvi19g04302\_t001 |  |  |  |  |  |  |  |  |  |  |  |  |
| 0 | Vitvi19g04303\_t001 |  |  |  |  |  |  |  |  |  |  |  |  |
| 0 | Vitvi19g04304\_t001 |  |  |  |  |  |  |  |  |  |  |  |  |
| 0 | Vitvi19g00835\_t001 |  |  |  |  |  |  |  |  |  |  |  |  |
| 0 | Vitvi19g04305\_t001 |  |  |  |  |  |  |  |  |  |  |  |  |
| 0 | Vitvi19g04306\_t001 |  |  |  |  |  |  |  |  |  |  |  |  |
| 0 | Vitvi19g00865\_t001 |  |  |  |  |  |  |  |  |  |  |  |  |
| 0 | Vitvi19g04307\_t001 |  |  |  |  |  |  |  |  |  |  |  |  |
| 0 | Vitvi19g02103\_t001 |  |  |  |  |  |  |  |  |  |  |  |  |
| 0 | Vitvi19g04308\_t001 |  |  |  |  |  |  |  |  |  |  |  |  |
| 0 | Vitvi19g00896\_t001 |  |  |  |  |  |  |  |  |  |  |  |  |
| 0 | Vitvi19g04309\_t001 |  |  |  |  |  |  |  |  |  |  |  |  |
| 0 | Vitvi19g02106\_t001 |  |  |  |  |  |  |  |  |  |  |  |  |
| 0 | Vitvi19g02108\_t001 |  |  |  |  |  |  |  |  |  |  |  |  |
| 0 | Vitvi19g00899\_t001 |  |  |  |  |  |  |  |  |  |  |  |  |
| 0 | Vitvi19g00900\_t001 |  |  |  |  |  |  |  |  |  |  |  |  |
| 0 | Vitvi19g04310\_t001 |  |  |  |  |  |  |  |  |  |  |  |  |
| 0 | Vitvi19g04311\_t001 |  |  |  |  |  |  |  |  |  |  |  |  |
| 0 | Vitvi19g02111\_t001 |  |  |  |  |  |  |  |  |  |  |  |  |
| 0 | Vitvi19g00904\_t001 |  |  |  |  |  |  |  |  |  |  |  |  |
| 0 | Vitvi19g00905\_t001 |  |  |  |  |  |  |  |  |  |  |  |  |
| 0 | Vitvi19g00906\_t001 |  |  |  |  |  |  |  |  |  |  |  |  |
| 0 | Vitvi19g00907\_t001 |  |  |  |  |  |  |  |  |  |  |  |  |
| 0 | Vitvi19g00908\_t001 |  |  |  |  |  |  |  |  |  |  |  |  |
| 0 | Vitvi19g00909\_t001 |  |  |  |  |  |  |  |  |  |  |  |  |
| 0 | Vitvi19g04312\_t001 |  |  |  |  |  |  |  |  |  |  |  |  |
| 0 | Vitvi19g00911\_t001 |  |  |  |  |  |  |  |  |  |  |  |  |
| 0 | Vitvi19g00912\_t001 |  |  |  |  |  |  |  |  |  |  |  |  |
| 0 | Vitvi19g00913\_t001 |  |  |  |  |  |  |  |  |  |  |  |  |
| 0 | Vitvi19g00915\_t001 |  |  |  |  |  |  |  |  |  |  |  |  |
| 0 | Vitvi19g00916\_t001 |  |  |  |  |  |  |  |  |  |  |  |  |
| 0 | Vitvi19g00917\_t003 |  |  |  |  |  |  |  |  |  |  |  |  |
| 0 | Vitvi19g02112\_t003 |  |  |  |  |  |  |  |  |  |  |  |  |
| 0 | Vitvi19g00919\_t001 |  |  |  |  |  |  |  |  |  |  |  |  |
| 0 | Vitvi19g00922\_t002 |  |  |  |  |  |  |  |  |  |  |  |  |
| 0 | Vitvi19g00927\_t001 |  |  |  |  |  |  |  |  |  |  |  |  |
| 0 | Vitvi19g00928\_t001 |  |  |  |  |  |  |  |  |  |  |  |  |
| 0 | Vitvi19g00929\_t001 |  |  |  |  |  |  |  |  |  |  |  |  |
| 0 | Vitvi19g00930\_t001 |  |  |  |  |  |  |  |  |  |  |  |  |
| 0 | Vitvi19g00931\_t001 |  |  |  |  |  |  |  |  |  |  |  |  |
| 0 | Vitvi19g00932\_t001 |  |  |  |  |  |  |  |  |  |  |  |  |
| 0 | Vitvi19g00933\_t001 |  |  |  |  |  |  |  |  |  |  |  |  |
| 0 | Vitvi19g04313\_t001 |  |  |  |  |  |  |  |  |  |  |  |  |
| 0 | Vitvi19g04314\_t001 |  |  |  |  |  |  |  |  |  |  |  |  |
| 0 | Vitvi19g04315\_t001 |  |  |  |  |  |  |  |  |  |  |  |  |
| 0 | Vitvi19g04316\_t001 |  |  |  |  |  |  |  |  |  |  |  |  |
| 0 | Vitvi19g00935\_t001 |  |  |  |  |  |  |  |  |  |  |  |  |
| 0 | Vitvi19g04317\_t001 |  |  |  |  |  |  |  |  |  |  |  |  |
| 0 | Vitvi19g04318\_t001 |  |  |  |  |  |  |  |  |  |  |  |  |
| 0 | Vitvi19g00937\_t001 |  |  |  |  |  |  |  |  |  |  |  |  |
| 0 | Vitvi19g02114\_t001 |  |  |  |  |  |  |  |  |  |  |  |  |
| 0 | Vitvi19g02116\_t001 |  |  |  |  |  |  |  |  |  |  |  |  |
| 0 | Vitvi19g02117\_t001 |  |  |  |  |  |  |  |  |  |  |  |  |
| 0 | Vitvi19g00940\_t001 |  |  |  |  |  |  |  |  |  |  |  |  |
| 0 | Vitvi19g04319\_t001 |  |  |  |  |  |  |  |  |  |  |  |  |
| 0 | Vitvi19g02119\_t001 |  |  |  |  |  |  |  |  |  |  |  |  |
| 0 | Vitvi19g04320\_t001 |  |  |  |  |  |  |  |  |  |  |  |  |
| 0 | Vitvi19g04321\_t001 |  |  |  |  |  |  |  |  |  |  |  |  |
| 0 | Vitvi19g04322\_t001 |  |  |  |  |  |  |  |  |  |  |  |  |
| 0 | Vitvi19g04323\_t001 |  |  |  |  |  |  |  |  |  |  |  |  |
| 0 | Vitvi19g02122\_t001 |  |  |  |  |  |  |  |  |  |  |  |  |
| 0 | Vitvi19g02123\_t001 |  |  |  |  |  |  |  |  |  |  |  |  |
| 0 | Vitvi19g04324\_t001 |  |  |  |  |  |  |  |  |  |  |  |  |
| 0 | Vitvi19g04325\_t001 |  |  |  |  |  |  |  |  |  |  |  |  |
| 0 | Vitvi19g00953\_t001 |  |  |  |  |  |  |  |  |  |  |  |  |
| 1 | Vitvi19g00954\_t001 |  | Vitvi19g01028\_t001 |  |  |  |  |  |  |  |  |  |  |
| 1 | Vitvi19g00956\_t002 |  | \| \| |  |  |  |  |  |  |  |  |  |  |
| 1 | Vitvi19g00957\_t001 |  | \| \| |  |  |  |  |  |  |  |  |  |  |
| 2 | Vitvi19g00958\_t001 |  | Vitvi19g04376\_t001 |  | Vitvi19g02212\_t001 |  |  |  |  |  |  |  |  |
| 2 | Vitvi19g02126\_t001 |  | \| \| |  | \| \| |  |  |  |  |  |  |  |  |
| 2 | Vitvi19g02127\_t001 |  | \| \| |  | \| \| |  |  |  |  |  |  |  |  |
| 2 | Vitvi19g04326\_t001 |  | \| \| |  | \| \| |  |  |  |  |  |  |  |  |
| 2 | Vitvi19g04327\_t001 |  | \| \| |  | \| \| |  |  |  |  |  |  |  |  |
| 2 | Vitvi19g00961\_t001 |  | \| \| |  | \| \| |  |  |  |  |  |  |  |  |
| 2 | Vitvi19g00962\_t001 |  | Vitvi19g04389\_t001 |  | Vitvi19g04393\_t001 |  |  |  |  |  |  |  |  |
| 2 | Vitvi19g04328\_t001 |  | \| \| |  | \| \| |  |  |  |  |  |  |  |  |
| 2 | Vitvi19g04329\_t001 |  | \| \| |  | \| \| |  |  |  |  |  |  |  |  |
| 2 | Vitvi19g04330\_t001 |  | \| \| |  | \| \| |  |  |  |  |  |  |  |  |
| 2 | Vitvi19g04331\_t001 |  | \| \| |  | \| \| |  |  |  |  |  |  |  |  |
| 2 | Vitvi19g04332\_t001 |  | \| \| |  | \| \| |  |  |  |  |  |  |  |  |
| 2 | Vitvi19g04333\_t001 |  | \| \| |  | \| \| |  |  |  |  |  |  |  |  |
| 2 | Vitvi19g04334\_t001 |  | \| \| |  | \| \| |  |  |  |  |  |  |  |  |
| 2 | Vitvi19g04335\_t001 |  | \| \| |  | \| \| |  |  |  |  |  |  |  |  |
| 2 | Vitvi19g04336\_t001 |  | \| \| |  | \| \| |  |  |  |  |  |  |  |  |
| 2 | Vitvi19g02130\_t001 |  | Vitvi19g04392\_t001 |  | \| \| |  |  |  |  |  |  |  |  |
| 2 | Vitvi19g04337\_t001 |  | \| \| |  | \| \| |  |  |  |  |  |  |  |  |
| 2 | Vitvi19g04338\_t001 |  | \| \| |  | \| \| |  |  |  |  |  |  |  |  |
| 2 | Vitvi19g00974\_t001 |  | \| \| |  | \| \| |  |  |  |  |  |  |  |  |
| 2 | Vitvi19g00975\_t001 |  | \| \| |  | Vitvi19g01371\_t001 |  |  |  |  |  |  |  |  |
| 2 | Vitvi19g00976\_t001 |  | \| \| |  | \| \| |  |  |  |  |  |  |  |  |
| 2 | Vitvi19g00978\_t001 |  | Vitvi19g02208\_t001 |  | Vitvi19g02208\_t001 |  |  |  |  |  |  |  |  |
| 2 | Vitvi19g04339\_t001 |  | \| \| |  | Vitvi19g04392\_t001 |  |  |  |  |  |  |  |  |
| 2 | Vitvi19g02133\_t001 |  | \| \| |  | \| \| |  |  |  |  |  |  |  |  |
| 2 | Vitvi19g00980\_t001 |  | \| \| |  | \| \| |  |  |  |  |  |  |  |  |
| 2 | Vitvi19g02135\_t001 |  | \| \| |  | \| \| |  |  |  |  |  |  |  |  |
| 2 | Vitvi19g04340\_t001 |  | \| \| |  | \| \| |  |  |  |  |  |  |  |  |
| 2 | Vitvi19g04341\_t001 |  | \| \| |  | \| \| |  |  |  |  |  |  |  |  |
| 2 | Vitvi19g04342\_t001 |  | \| \| |  | \| \| |  |  |  |  |  |  |  |  |
| 2 | Vitvi19g04343\_t001 |  | \| \| |  | \| \| |  |  |  |  |  |  |  |  |
| 2 | Vitvi19g02136\_t001 |  | \| \| |  | \| \| |  |  |  |  |  |  |  |  |
| 2 | Vitvi19g00986\_t001 |  | \| \| |  | \| \| |  |  |  |  |  |  |  |  |
| 2 | Vitvi19g00987\_t001 |  | \| \| |  | \| \| |  |  |  |  |  |  |  |  |
| 2 | Vitvi19g04344\_t001 |  | \| \| |  | \| \| |  |  |  |  |  |  |  |  |
| 2 | Vitvi19g04345\_t001 |  | \| \| |  | \| \| |  |  |  |  |  |  |  |  |
| 2 | Vitvi19g04346\_t001 |  | \| \| |  | \| \| |  |  |  |  |  |  |  |  |
| 2 | Vitvi19g00991\_t001 |  | \| \| |  | \| \| |  |  |  |  |  |  |  |  |
| 2 | Vitvi19g04347\_t001 |  | \| \| |  | \| \| |  |  |  |  |  |  |  |  |
| 2 | Vitvi19g04348\_t001 |  | \| \| |  | \| \| |  |  |  |  |  |  |  |  |
| 2 | Vitvi19g00994\_t001 |  | \| \| |  | \| \| |  |  |  |  |  |  |  |  |
| 2 | Vitvi19g04349\_t001 |  | \| \| |  | \| \| |  |  |  |  |  |  |  |  |
| 2 | Vitvi19g04350\_t001 |  | \| \| |  | \| \| |  |  |  |  |  |  |  |  |
| 2 | Vitvi19g04351\_t001 |  | \| \| |  | \| \| |  |  |  |  |  |  |  |  |
| 2 | Vitvi19g04352\_t001 |  | \| \| |  | \| \| |  |  |  |  |  |  |  |  |
| 2 | Vitvi19g02140\_t001 |  | \| \| |  | Vitvi19g04389\_t001 |  |  |  |  |  |  |  |  |
| 2 | Vitvi19g04353\_t001 |  | Vitvi19g01371\_t001 |  | \| \| |  |  |  |  |  |  |  |  |
| 2 | Vitvi19g04354\_t001 |  | \| \| |  | \| \| |  |  |  |  |  |  |  |  |
| 2 | Vitvi19g04355\_t001 |  | \| \| |  | \| \| |  |  |  |  |  |  |  |  |
| 2 | Vitvi19g04356\_t001 |  | \| \| |  | \| \| |  |  |  |  |  |  |  |  |
| 2 | Vitvi19g04357\_t001 |  | \| \| |  | \| \| |  |  |  |  |  |  |  |  |
| 2 | Vitvi19g04358\_t001 |  | \| \| |  | \| \| |  |  |  |  |  |  |  |  |
| 2 | Vitvi19g01000\_t001 |  | \| \| |  | \| \| |  |  |  |  |  |  |  |  |
| 2 | Vitvi19g01001\_t001 |  | \| \| |  | \| \| |  |  |  |  |  |  |  |  |
| 2 | Vitvi19g04359\_t001 |  | \| \| |  | \| \| |  |  |  |  |  |  |  |  |
| 2 | Vitvi19g01003\_t001 |  | \| \| |  | \| \| |  |  |  |  |  |  |  |  |
| 2 | Vitvi19g02141\_t002 |  | \| \| |  | \| \| |  |  |  |  |  |  |  |  |
| 2 | Vitvi19g01005\_t001 |  | Vitvi19g04395\_t001 |  | Vitvi19g04387\_t001 |  |  |  |  |  |  |  |  |
| 1 | Vitvi19g04360\_t001 |  |  |  | \| \| |  |  |  |  |  |  |  |  |
| 1 | Vitvi19g04361\_t001 |  |  |  | Vitvi19g04373\_t001 |  |  |  |  |  |  |  |  |
| 0 | Vitvi19g04362\_t001 |  |  |  |  |  |  |  |  |  |  |  |  |
| 0 | Vitvi19g04363\_t001 |  |  |  |  |  |  |  |  |  |  |  |  |
| 0 | Vitvi19g04364\_t001 |  |  |  |  |  |  |  |  |  |  |  |  |
| 0 | Vitvi19g04365\_t001 |  |  |  |  |  |  |  |  |  |  |  |  |
| 0 | Vitvi19g04366\_t001 |  |  |  |  |  |  |  |  |  |  |  |  |
| 0 | Vitvi19g04367\_t001 |  |  |  |  |  |  |  |  |  |  |  |  |
| 1 | Vitvi19g01028\_t001 |  | Vitvi19g00954\_t001 |  |  |  |  |  |  |  |  |  |  |
| 1 | Vitvi19g04368\_t001 |  | \| \| |  |  |  |  |  |  |  |  |  |  |
| 1 | Vitvi19g04369\_t001 |  | \| \| |  |  |  |  |  |  |  |  |  |  |
| 1 | Vitvi19g04370\_t001 |  | \| \| |  |  |  |  |  |  |  |  |  |  |
| 1 | Vitvi19g04371\_t001 |  | \| \| |  |  |  |  |  |  |  |  |  |  |
| 1 | Vitvi19g04372\_t001 |  | \| \| |  |  |  |  |  |  |  |  |  |  |
| 2 | Vitvi19g04373\_t001 |  | \| \| |  | Vitvi19g04361\_t001 |  |  |  |  |  |  |  |  |
| 2 | Vitvi19g04374\_t001 |  | \| \| |  | \| \| |  |  |  |  |  |  |  |  |
| 2 | Vitvi19g04375\_t001 |  | \| \| |  | \| \| |  |  |  |  |  |  |  |  |
| 2 | Vitvi19g04376\_t001 |  | Vitvi19g00958\_t001 |  | \| \| |  |  |  |  |  |  |  |  |
| 2 | Vitvi19g02157\_t001 |  | \| \| |  | \| \| |  |  |  |  |  |  |  |  |
| 2 | Vitvi19g04377\_t001 |  | \| \| |  | \| \| |  |  |  |  |  |  |  |  |
| 2 | Vitvi19g04378\_t001 |  | \| \| |  | \| \| |  |  |  |  |  |  |  |  |
| 2 | Vitvi19g04379\_t001 |  | \| \| |  | \| \| |  |  |  |  |  |  |  |  |
| 2 | Vitvi19g04380\_t001 |  | \| \| |  | \| \| |  |  |  |  |  |  |  |  |
| 2 | Vitvi19g04381\_t001 |  | \| \| |  | \| \| |  |  |  |  |  |  |  |  |
| 2 | Vitvi19g04383\_t001 |  | \| \| |  | \| \| |  |  |  |  |  |  |  |  |
| 2 | Vitvi19g04384\_t001 |  | \| \| |  | \| \| |  |  |  |  |  |  |  |  |
| 2 | Vitvi19g04385\_t001 |  | \| \| |  | \| \| |  |  |  |  |  |  |  |  |
| 2 | Vitvi19g04386\_t001 |  | \| \| |  | \| \| |  |  |  |  |  |  |  |  |
| 2 | Vitvi19g04387\_t001 |  | \| \| |  | Vitvi19g01005\_t001 |  |  |  |  |  |  |  |  |
| 2 | Vitvi19g01058\_t001 |  | \| \| |  | \| \| |  |  |  |  |  |  |  |  |
| 2 | Vitvi19g04388\_t001 |  | \| \| |  | \| \| |  |  |  |  |  |  |  |  |
| 2 | Vitvi19g02164\_t001 |  | \| \| |  | \| \| |  |  |  |  |  |  |  |  |
| 2 | Vitvi19g04389\_t001 |  | Vitvi19g00962\_t001 |  | Vitvi19g02140\_t001 |  |  |  |  |  |  |  |  |
| 2 | Vitvi19g02165\_t001 |  | \| \| |  | \| \| |  |  |  |  |  |  |  |  |
| 2 | Vitvi19g04391\_t001 |  | \| \| |  | \| \| |  |  |  |  |  |  |  |  |
| 2 | Vitvi19g04392\_t001 |  | Vitvi19g02130\_t001 |  | Vitvi19g04339\_t001 |  |  |  |  |  |  |  |  |
| 2 | Vitvi19g02208\_t001 |  | Vitvi19g00978\_t001 |  | Vitvi19g00978\_t001 |  |  |  |  |  |  |  |  |
| 2 | Vitvi19g01371\_t001 |  | Vitvi19g04353\_t001 |  | Vitvi19g00975\_t001 |  |  |  |  |  |  |  |  |
| 2 | Vitvi19g04393\_t001 |  | \| \| |  | Vitvi19g00962\_t001 |  |  |  |  |  |  |  |  |
| 2 | Vitvi19g04394\_t001 |  | \| \| |  | \| \| |  |  |  |  |  |  |  |  |
| 2 | Vitvi19g01375\_t001 |  | \| \| |  | \| \| |  |  |  |  |  |  |  |  |
| 2 | Vitvi19g04395\_t001 |  | Vitvi19g01005\_t001 |  | \| \| |  |  |  |  |  |  |  |  |
| 1 | Vitvi19g04396\_t001 |  |  |  | \| \| |  |  |  |  |  |  |  |  |
| 1 | Vitvi19g01381\_t001 |  |  |  | \| \| |  |  |  |  |  |  |  |  |
| 1 | Vitvi19g04397\_t001 |  |  |  | \| \| |  |  |  |  |  |  |  |  |
| 1 | Vitvi19g04398\_t001 |  |  |  | \| \| |  |  |  |  |  |  |  |  |
| 1 | Vitvi19g04399\_t001 |  |  |  | \| \| |  |  |  |  |  |  |  |  |
| 1 | Vitvi19g01383\_t001 |  |  |  | \| \| |  |  |  |  |  |  |  |  |
| 1 | Vitvi19g02212\_t001 |  |  |  | Vitvi19g00958\_t001 |  |  |  |  |  |  |  |  |
| 0 | Vitvi19g04400\_t001 |  |  |  |  |  |  |  |  |  |  |  |  |
| 0 | Vitvi19g04401\_t001 |  |  |  |  |  |  |  |  |  |  |  |  |
| 0 | Vitvi19g02216\_t001 |  |  |  |  |  |  |  |  |  |  |  |  |
| 0 | Vitvi19g04402\_t001 |  |  |  |  |  |  |  |  |  |  |  |  |
| 0 | Vitvi19g04403\_t001 |  |  |  |  |  |  |  |  |  |  |  |  |
| 0 | Vitvi19g01389\_t001 |  |  |  |  |  |  |  |  |  |  |  |  |
| 0 | Vitvi19g04404\_t001 |  |  |  |  |  |  |  |  |  |  |  |  |
| 0 | Vitvi19g01391\_t001 |  |  |  |  |  |  |  |  |  |  |  |  |
| 0 | Vitvi19g01394\_t001 |  |  |  |  |  |  |  |  |  |  |  |  |
| 0 | Vitvi19g01400\_t001 |  |  |  |  |  |  |  |  |  |  |  |  |
| 0 | Vitvi19g01403\_t001 |  |  |  |  |  |  |  |  |  |  |  |  |
| 0 | Vitvi19g04405\_t001 |  |  |  |  |  |  |  |  |  |  |  |  |
| 0 | Vitvi19g01445\_t001 |  |  |  |  |  |  |  |  |  |  |  |  |
| 0 | Vitvi19g04406\_t001 |  |  |  |  |  |  |  |  |  |  |  |  |
| 0 | Vitvi19g01442\_t001 |  |  |  |  |  |  |  |  |  |  |  |  |
| 0 | Vitvi19g04407\_t001 |  |  |  |  |  |  |  |  |  |  |  |  |
| 0 | Vitvi19g04408\_t001 |  |  |  |  |  |  |  |  |  |  |  |  |
| 0 | Vitvi19g04409\_t001 |  |  |  |  |  |  |  |  |  |  |  |  |
| 0 | Vitvi19g04410\_t001 |  |  |  |  |  |  |  |  |  |  |  |  |
| 0 | Vitvi19g02236\_t001 |  |  |  |  |  |  |  |  |  |  |  |  |
| 0 | Vitvi19g01440\_t001 |  |  |  |  |  |  |  |  |  |  |  |  |
| 0 | Vitvi19g01438\_t001 |  |  |  |  |  |  |  |  |  |  |  |  |
| 0 | Vitvi19g04411\_t001 |  |  |  |  |  |  |  |  |  |  |  |  |
| 0 | Vitvi19g02232\_t001 |  |  |  |  |  |  |  |  |  |  |  |  |
| 0 | Vitvi19g04412\_t001 |  |  |  |  |  |  |  |  |  |  |  |  |
| 0 | Vitvi19g04413\_t001 |  |  |  |  |  |  |  |  |  |  |  |  |
| 0 | Vitvi19g01432\_t001 |  |  |  |  |  |  |  |  |  |  |  |  |
| 0 | Vitvi19g02230\_t001 |  |  |  |  |  |  |  |  |  |  |  |  |
| 0 | Vitvi19g04414\_t001 |  |  |  |  |  |  |  |  |  |  |  |  |
| 0 | Vitvi19g01426\_t001 |  |  |  |  |  |  |  |  |  |  |  |  |
| 0 | Vitvi19g01425\_t001 |  |  |  |  |  |  |  |  |  |  |  |  |
| 0 | Vitvi19g04415\_t001 |  |  |  |  |  |  |  |  |  |  |  |  |
| 0 | Vitvi19g04416\_t001 |  |  |  |  |  |  |  |  |  |  |  |  |
| 0 | Vitvi19g04417\_t001 |  |  |  |  |  |  |  |  |  |  |  |  |
| 0 | Vitvi19g04418\_t001 |  |  |  |  |  |  |  |  |  |  |  |  |
| 0 | Vitvi19g04419\_t001 |  |  |  |  |  |  |  |  |  |  |  |  |
| 0 | Vitvi19g04420\_t001 |  |  |  |  |  |  |  |  |  |  |  |  |
| 0 | Vitvi19g04421\_t001 |  |  |  |  |  |  |  |  |  |  |  |  |
| 0 | Vitvi19g01420\_t001 |  |  |  |  |  |  |  |  |  |  |  |  |
| 0 | Vitvi19g04422\_t001 |  |  |  |  |  |  |  |  |  |  |  |  |
| 0 | Vitvi19g04423\_t001 |  |  |  |  |  |  |  |  |  |  |  |  |
| 0 | Vitvi19g04424\_t001 |  |  |  |  |  |  |  |  |  |  |  |  |
| 0 | Vitvi19g04425\_t001 |  |  |  |  |  |  |  |  |  |  |  |  |
| 0 | Vitvi19g04426\_t001 |  |  |  |  |  |  |  |  |  |  |  |  |
| 0 | Vitvi19g01276\_t001 |  |  |  |  |  |  |  |  |  |  |  |  |
| 0 | Vitvi19g04427\_t001 |  |  |  |  |  |  |  |  |  |  |  |  |
| 0 | Vitvi19g04428\_t001 |  |  |  |  |  |  |  |  |  |  |  |  |
| 0 | Vitvi19g02178\_t001 |  |  |  |  |  |  |  |  |  |  |  |  |
| 0 | Vitvi19g01277\_t001 |  |  |  |  |  |  |  |  |  |  |  |  |
| 0 | Vitvi19g04429\_t001 |  |  |  |  |  |  |  |  |  |  |  |  |
| 0 | Vitvi19g01278\_t001 |  |  |  |  |  |  |  |  |  |  |  |  |
| 0 | Vitvi19g01281\_t001 |  |  |  |  |  |  |  |  |  |  |  |  |
| 0 | Vitvi19g02180\_t001 |  |  |  |  |  |  |  |  |  |  |  |  |
| 0 | Vitvi19g04430\_t001 |  |  |  |  |  |  |  |  |  |  |  |  |
| 0 | Vitvi19g01284\_t001 |  |  |  |  |  |  |  |  |  |  |  |  |
| 0 | Vitvi19g04431\_t001 |  |  |  |  |  |  |  |  |  |  |  |  |
| 0 | Vitvi19g04432\_t001 |  |  |  |  |  |  |  |  |  |  |  |  |
| 0 | Vitvi19g01415\_t001 |  |  |  |  |  |  |  |  |  |  |  |  |
| 0 | Vitvi19g04433\_t001 |  |  |  |  |  |  |  |  |  |  |  |  |
| 0 | Vitvi19g04434\_t001 |  |  |  |  |  |  |  |  |  |  |  |  |
| 0 | Vitvi19g02222\_t001 |  |  |  |  |  |  |  |  |  |  |  |  |
| 0 | Vitvi19g04435\_t001 |  |  |  |  |  |  |  |  |  |  |  |  |
| 0 | Vitvi19g04436\_t001 |  |  |  |  |  |  |  |  |  |  |  |  |
| 0 | Vitvi19g01408\_t001 |  |  |  |  |  |  |  |  |  |  |  |  |
| 0 | Vitvi19g04437\_t001 |  |  |  |  |  |  |  |  |  |  |  |  |
| 0 | Vitvi19g04438\_t001 |  |  |  |  |  |  |  |  |  |  |  |  |
| 0 | Vitvi19g04439\_t001 |  |  |  |  |  |  |  |  |  |  |  |  |
| 0 | Vitvi19g01368\_t001 |  |  |  |  |  |  |  |  |  |  |  |  |
| 0 | Vitvi19g04440\_t001 |  |  |  |  |  |  |  |  |  |  |  |  |
| 0 | Vitvi19g04441\_t001 |  |  |  |  |  |  |  |  |  |  |  |  |
| 0 | Vitvi19g04442\_t001 |  |  |  |  |  |  |  |  |  |  |  |  |
| 0 | Vitvi19g01363\_t001 |  |  |  |  |  |  |  |  |  |  |  |  |
| 0 | Vitvi19g01360\_t001 |  |  |  |  |  |  |  |  |  |  |  |  |
| 0 | Vitvi19g01359\_t003 |  |  |  |  |  |  |  |  |  |  |  |  |
| 0 | Vitvi19g01357\_t001 |  |  |  |  |  |  |  |  |  |  |  |  |
| 0 | Vitvi19g01356\_t001 |  |  |  |  |  |  |  |  |  |  |  |  |
| 0 | Vitvi19g04443\_t001 |  |  |  |  |  |  |  |  |  |  |  |  |
| 0 | Vitvi19g01352\_t001 |  |  |  |  |  |  |  |  |  |  |  |  |
| 0 | Vitvi19g04444\_t001 |  |  |  |  |  |  |  |  |  |  |  |  |
| 0 | Vitvi19g01351\_t001 |  |  |  |  |  |  |  |  |  |  |  |  |
| 0 | Vitvi19g01349\_t001 |  |  |  |  |  |  |  |  |  |  |  |  |
| 0 | Vitvi19g01346\_t001 |  |  |  |  |  |  |  |  |  |  |  |  |
| 0 | Vitvi19g04445\_t001 |  |  |  |  |  |  |  |  |  |  |  |  |
| 0 | Vitvi19g01345\_t001 |  |  |  |  |  |  |  |  |  |  |  |  |
| 0 | Vitvi19g04446\_t001 |  |  |  |  |  |  |  |  |  |  |  |  |
| 0 | Vitvi19g04447\_t001 |  |  |  |  |  |  |  |  |  |  |  |  |
| 0 | Vitvi19g04448\_t001 |  |  |  |  |  |  |  |  |  |  |  |  |
| 0 | Vitvi19g02201\_t001 |  |  |  |  |  |  |  |  |  |  |  |  |
| 0 | Vitvi19g04449\_t001 |  |  |  |  |  |  |  |  |  |  |  |  |
| 0 | Vitvi19g01339\_t001 |  |  |  |  |  |  |  |  |  |  |  |  |
| 0 | Vitvi19g02200\_t001 |  |  |  |  |  |  |  |  |  |  |  |  |
| 0 | Vitvi19g04450\_t001 |  |  |  |  |  |  |  |  |  |  |  |  |
| 0 | Vitvi19g01335\_t001 |  |  |  |  |  |  |  |  |  |  |  |  |
| 0 | Vitvi19g01332\_t001 |  |  |  |  |  |  |  |  |  |  |  |  |
| 0 | Vitvi19g04451\_t001 |  |  |  |  |  |  |  |  |  |  |  |  |
| 0 | Vitvi19g02198\_t001 |  |  |  |  |  |  |  |  |  |  |  |  |
| 0 | Vitvi19g04452\_t001 |  |  |  |  |  |  |  |  |  |  |  |  |
| 0 | Vitvi19g04453\_t001 |  |  |  |  |  |  |  |  |  |  |  |  |
| 0 | Vitvi19g04454\_t001 |  |  |  |  |  |  |  |  |  |  |  |  |
| 0 | Vitvi19g04455\_t001 |  |  |  |  |  |  |  |  |  |  |  |  |
| 0 | Vitvi19g04456\_t001 |  |  |  |  |  |  |  |  |  |  |  |  |
| 0 | Vitvi19g04457\_t001 |  |  |  |  |  |  |  |  |  |  |  |  |
| 0 | Vitvi19g01316\_t001 |  |  |  |  |  |  |  |  |  |  |  |  |
| 0 | Vitvi19g04458\_t001 |  |  |  |  |  |  |  |  |  |  |  |  |
| 0 | Vitvi19g01309\_t001 |  |  |  |  |  |  |  |  |  |  |  |  |
| 0 | Vitvi19g04459\_t001 |  |  |  |  |  |  |  |  |  |  |  |  |
| 0 | Vitvi19g02193\_t001 |  |  |  |  |  |  |  |  |  |  |  |  |
| 0 | Vitvi19g01307\_t001 |  |  |  |  |  |  |  |  |  |  |  |  |
| 0 | Vitvi19g01302\_t001 |  |  |  |  |  |  |  |  |  |  |  |  |
| 0 | Vitvi19g02188\_t001 |  |  |  |  |  |  |  |  |  |  |  |  |
| 0 | Vitvi19g01299\_t001 |  |  |  |  |  |  |  |  |  |  |  |  |
| 0 | Vitvi19g04460\_t001 |  |  |  |  |  |  |  |  |  |  |  |  |
| 0 | Vitvi19g04461\_t001 |  |  |  |  |  |  |  |  |  |  |  |  |
| 0 | Vitvi19g04462\_t001 |  |  |  |  |  |  |  |  |  |  |  |  |
| 0 | Vitvi19g04463\_t001 |  |  |  |  |  |  |  |  |  |  |  |  |
| 0 | Vitvi19g02186\_t001 |  |  |  |  |  |  |  |  |  |  |  |  |
| 0 | Vitvi19g02185\_t001 |  |  |  |  |  |  |  |  |  |  |  |  |
| 0 | Vitvi19g04464\_t001 |  |  |  |  |  |  |  |  |  |  |  |  |
| 0 | Vitvi19g04465\_t001 |  |  |  |  |  |  |  |  |  |  |  |  |
| 0 | Vitvi19g04466\_t001 |  |  |  |  |  |  |  |  |  |  |  |  |
| 0 | Vitvi19g01446\_t001 |  |  |  |  |  |  |  |  |  |  |  |  |
| 0 | Vitvi19g04467\_t001 |  |  |  |  |  |  |  |  |  |  |  |  |
| 0 | Vitvi19g01450\_t001 |  |  |  |  |  |  |  |  |  |  |  |  |
| 0 | Vitvi19g04468\_t001 |  |  |  |  |  |  |  |  |  |  |  |  |
| 0 | Vitvi19g04469\_t001 |  |  |  |  |  |  |  |  |  |  |  |  |
| 0 | Vitvi19g04470\_t001 |  |  |  |  |  |  |  |  |  |  |  |  |
| 0 | Vitvi19g04471\_t001 |  |  |  |  |  |  |  |  |  |  |  |  |
| 0 | Vitvi19g01455\_t001 |  |  |  |  |  |  |  |  |  |  |  |  |
| 0 | Vitvi19g01456\_t001 |  |  |  |  |  |  |  |  |  |  |  |  |
| 0 | Vitvi19g01457\_t001 |  |  |  |  |  |  |  |  |  |  |  |  |
| 0 | Vitvi19g04472\_t001 |  |  |  |  |  |  |  |  |  |  |  |  |
| 0 | Vitvi19g04473\_t001 |  |  |  |  |  |  |  |  |  |  |  |  |
| 0 | Vitvi19g01459\_t001 |  |  |  |  |  |  |  |  |  |  |  |  |
| 0 | Vitvi19g01462\_t001 |  |  |  |  |  |  |  |  |  |  |  |  |
| 0 | Vitvi19g04474\_t001 |  |  |  |  |  |  |  |  |  |  |  |  |
| 0 | Vitvi19g04475\_t001 |  |  |  |  |  |  |  |  |  |  |  |  |
| 0 | Vitvi19g04476\_t001 |  |  |  |  |  |  |  |  |  |  |  |  |
| 0 | Vitvi19g04477\_t001 |  |  |  |  |  |  |  |  |  |  |  |  |
| 0 | Vitvi19g01463\_t001 |  |  |  |  |  |  |  |  |  |  |  |  |
| 0 | Vitvi19g01464\_t001 |  |  |  |  |  |  |  |  |  |  |  |  |
| 0 | Vitvi19g01465\_t001 |  |  |  |  |  |  |  |  |  |  |  |  |
| 0 | Vitvi19g04478\_t001 |  |  |  |  |  |  |  |  |  |  |  |  |
| 0 | Vitvi19g04479\_t001 |  |  |  |  |  |  |  |  |  |  |  |  |
| 0 | Vitvi19g01471\_t001 |  |  |  |  |  |  |  |  |  |  |  |  |
| 0 | Vitvi19g04480\_t001 |  |  |  |  |  |  |  |  |  |  |  |  |
| 0 | Vitvi19g01473\_t001 |  |  |  |  |  |  |  |  |  |  |  |  |
| 0 | Vitvi19g04481\_t001 |  |  |  |  |  |  |  |  |  |  |  |  |
| 0 | Vitvi19g04482\_t001 |  |  |  |  |  |  |  |  |  |  |  |  |
| 0 | Vitvi19g01477\_t001 |  |  |  |  |  |  |  |  |  |  |  |  |
| 0 | Vitvi19g01479\_t001 |  |  |  |  |  |  |  |  |  |  |  |  |
| 0 | Vitvi19g01480\_t001 |  |  |  |  |  |  |  |  |  |  |  |  |
| 0 | Vitvi19g04483\_t001 |  |  |  |  |  |  |  |  |  |  |  |  |
| 0 | Vitvi19g01483\_t001 |  |  |  |  |  |  |  |  |  |  |  |  |
| 0 | Vitvi19g01484\_t001 |  |  |  |  |  |  |  |  |  |  |  |  |
| 0 | Vitvi19g01486\_t001 |  |  |  |  |  |  |  |  |  |  |  |  |
| 0 | Vitvi19g01487\_t001 |  |  |  |  |  |  |  |  |  |  |  |  |
| 0 | Vitvi19g04484\_t001 |  |  |  |  |  |  |  |  |  |  |  |  |
| 0 | Vitvi19g04485\_t001 |  |  |  |  |  |  |  |  |  |  |  |  |
| 0 | Vitvi19g01785\_t001 |  |  |  |  |  |  |  |  |  |  |  |  |
| 0 | Vitvi19g01491\_t001 |  |  |  |  |  |  |  |  |  |  |  |  |
| 0 | Vitvi19g01493\_t001 |  |  |  |  |  |  |  |  |  |  |  |  |
| 0 | Vitvi19g04486\_t001 |  |  |  |  |  |  |  |  |  |  |  |  |
| 0 | Vitvi19g04487\_t001 |  |  |  |  |  |  |  |  |  |  |  |  |
| 0 | Vitvi19g04488\_t001 |  |  |  |  |  |  |  |  |  |  |  |  |
| 0 | Vitvi19g02251\_t001 |  |  |  |  |  |  |  |  |  |  |  |  |
| 0 | Vitvi19g04489\_t001 |  |  |  |  |  |  |  |  |  |  |  |  |
| 0 | Vitvi19g01499\_t001 |  |  |  |  |  |  |  |  |  |  |  |  |
| 0 | Vitvi19g02252\_t001 |  |  |  |  |  |  |  |  |  |  |  |  |
| 0 | Vitvi19g04490\_t001 |  |  |  |  |  |  |  |  |  |  |  |  |
| 0 | Vitvi19g01500\_t001 |  |  |  |  |  |  |  |  |  |  |  |  |
| 0 | Vitvi19g01501\_t002 |  |  |  |  |  |  |  |  |  |  |  |  |
| 0 | Vitvi19g04491\_t001 |  |  |  |  |  |  |  |  |  |  |  |  |
| 0 | Vitvi19g01503\_t001 |  |  |  |  |  |  |  |  |  |  |  |  |
| 0 | Vitvi19g01504\_t001 |  |  |  |  |  |  |  |  |  |  |  |  |
| 0 | Vitvi19g01505\_t001 |  |  |  |  |  |  |  |  |  |  |  |  |
| 0 | Vitvi19g04492\_t001 |  |  |  |  |  |  |  |  |  |  |  |  |
| 0 | Vitvi19g01507\_t001 |  |  |  |  |  |  |  |  |  |  |  |  |
| 0 | Vitvi19g04493\_t001 |  |  |  |  |  |  |  |  |  |  |  |  |
| 0 | Vitvi19g04494\_t001 |  |  |  |  |  |  |  |  |  |  |  |  |
| 0 | Vitvi19g04495\_t001 |  |  |  |  |  |  |  |  |  |  |  |  |
| 0 | Vitvi19g01512\_t001 |  |  |  |  |  |  |  |  |  |  |  |  |
| 0 | Vitvi19g01514\_t001 |  |  |  |  |  |  |  |  |  |  |  |  |
| 0 | Vitvi19g04496\_t001 |  |  |  |  |  |  |  |  |  |  |  |  |
| 0 | Vitvi19g01517\_t001 |  |  |  |  |  |  |  |  |  |  |  |  |
| 0 | Vitvi19g01518\_t001 |  |  |  |  |  |  |  |  |  |  |  |  |
| 0 | Vitvi19g02259\_t002 |  |  |  |  |  |  |  |  |  |  |  |  |
| 0 | Vitvi19g04497\_t001 |  |  |  |  |  |  |  |  |  |  |  |  |
| 0 | Vitvi19g04498\_t001 |  |  |  |  |  |  |  |  |  |  |  |  |
| 0 | Vitvi19g01522\_t001 |  |  |  |  |  |  |  |  |  |  |  |  |
| 0 | Vitvi19g01523\_t001 |  |  |  |  |  |  |  |  |  |  |  |  |
| 0 | Vitvi19g02261\_t001 |  |  |  |  |  |  |  |  |  |  |  |  |
| 0 | Vitvi19g01524\_t001 |  |  |  |  |  |  |  |  |  |  |  |  |
| 0 | Vitvi19g01525\_t001 |  |  |  |  |  |  |  |  |  |  |  |  |
| 0 | Vitvi19g04499\_t001 |  |  |  |  |  |  |  |  |  |  |  |  |
| 0 | Vitvi19g04500\_t001 |  |  |  |  |  |  |  |  |  |  |  |  |
| 0 | Vitvi19g04501\_t001 |  |  |  |  |  |  |  |  |  |  |  |  |
| 0 | Vitvi19g01530\_t001 |  |  |  |  |  |  |  |  |  |  |  |  |
| 0 | Vitvi19g01531\_t001 |  |  |  |  |  |  |  |  |  |  |  |  |
| 0 | Vitvi19g01532\_t001 |  |  |  |  |  |  |  |  |  |  |  |  |
| 0 | Vitvi19g01533\_t007 |  |  |  |  |  |  |  |  |  |  |  |  |
| 0 | Vitvi19g04502\_t001 |  |  |  |  |  |  |  |  |  |  |  |  |
| 0 | Vitvi19g04503\_t001 |  |  |  |  |  |  |  |  |  |  |  |  |
| 0 | Vitvi19g04504\_t001 |  |  |  |  |  |  |  |  |  |  |  |  |
| 0 | Vitvi19g04505\_t001 |  |  |  |  |  |  |  |  |  |  |  |  |
| 0 | Vitvi19g02263\_t001 |  |  |  |  |  |  |  |  |  |  |  |  |
| 0 | Vitvi19g04506\_t001 |  |  |  |  |  |  |  |  |  |  |  |  |
| 0 | Vitvi19g04507\_t001 |  |  |  |  |  |  |  |  |  |  |  |  |
| 1 | Vitvi19g04508\_t001 |  | Vitvi19g04581\_t001 |  |  |  |  |  |  |  |  |  |  |
| 1 | Vitvi19g04509\_t001 |  | \| \| |  |  |  |  |  |  |  |  |  |  |
| 1 | Vitvi19g04510\_t001 |  | \| \| |  |  |  |  |  |  |  |  |  |  |
| 1 | Vitvi19g01545\_t001 |  | \| \| |  |  |  |  |  |  |  |  |  |  |
| 1 | Vitvi19g04511\_t001 |  | \| \| |  |  |  |  |  |  |  |  |  |  |
| 1 | Vitvi19g04512\_t001 |  | \| \| |  |  |  |  |  |  |  |  |  |  |
| 1 | Vitvi19g02267\_t001 |  | \| \| |  |  |  |  |  |  |  |  |  |  |
| 1 | Vitvi19g04513\_t001 |  | \| \| |  |  |  |  |  |  |  |  |  |  |
| 1 | Vitvi19g01548\_t001 |  | \| \| |  |  |  |  |  |  |  |  |  |  |
| 1 | Vitvi19g01549\_t001 |  | \| \| |  |  |  |  |  |  |  |  |  |  |
| 1 | Vitvi19g04514\_t001 |  | \| \| |  |  |  |  |  |  |  |  |  |  |
| 1 | Vitvi19g01550\_t001 |  | \| \| |  |  |  |  |  |  |  |  |  |  |
| 1 | Vitvi19g01551\_t001 |  | \| \| |  |  |  |  |  |  |  |  |  |  |
| 1 | Vitvi19g01552\_t001 |  | \| \| |  |  |  |  |  |  |  |  |  |  |
| 1 | Vitvi19g02269\_t001 |  | \| \| |  |  |  |  |  |  |  |  |  |  |
| 1 | Vitvi19g01555\_t001 |  | \| \| |  |  |  |  |  |  |  |  |  |  |
| 1 | Vitvi19g01557\_t001 |  | \| \| |  |  |  |  |  |  |  |  |  |  |
| 1 | Vitvi19g01559\_t001 |  | \| \| |  |  |  |  |  |  |  |  |  |  |
| 1 | Vitvi19g04515\_t001 |  | \| \| |  |  |  |  |  |  |  |  |  |  |
| 1 | Vitvi19g04516\_t001 |  | \| \| |  |  |  |  |  |  |  |  |  |  |
| 1 | Vitvi19g04517\_t001 |  | Vitvi19g02300\_t001 |  |  |  |  |  |  |  |  |  |  |
| 1 | Vitvi19g02270\_t001 |  | Vitvi19g01610\_t001 |  |  |  |  |  |  |  |  |  |  |
| 1 | Vitvi19g01561\_t001 |  | \| \| |  |  |  |  |  |  |  |  |  |  |
| 1 | Vitvi19g04518\_t001 |  | \| \| |  |  |  |  |  |  |  |  |  |  |
| 1 | Vitvi19g02272\_t001 |  | \| \| |  |  |  |  |  |  |  |  |  |  |
| 1 | Vitvi19g02273\_t001 |  | \| \| |  |  |  |  |  |  |  |  |  |  |
| 1 | Vitvi19g01564\_t001 |  | \| \| |  |  |  |  |  |  |  |  |  |  |
| 1 | Vitvi19g01565\_t001 |  | Vitvi19g01586\_t001 |  |  |  |  |  |  |  |  |  |  |
| 1 | Vitvi19g04519\_t001 |  | \| \| |  |  |  |  |  |  |  |  |  |  |
| 1 | Vitvi19g04520\_t001 |  | \| \| |  |  |  |  |  |  |  |  |  |  |
| 1 | Vitvi19g01566\_t001 |  | \| \| |  |  |  |  |  |  |  |  |  |  |
| 1 | Vitvi19g04521\_t001 |  | \| \| |  |  |  |  |  |  |  |  |  |  |
| 1 | Vitvi19g04522\_t001 |  | \| \| |  |  |  |  |  |  |  |  |  |  |
| 1 | Vitvi19g04523\_t001 |  | \| \| |  |  |  |  |  |  |  |  |  |  |
| 1 | Vitvi19g04524\_t001 |  | \| \| |  |  |  |  |  |  |  |  |  |  |
| 1 | Vitvi19g04525\_t001 |  | \| \| |  |  |  |  |  |  |  |  |  |  |
| 1 | Vitvi19g04526\_t001 |  | \| \| |  |  |  |  |  |  |  |  |  |  |
| 1 | Vitvi19g04527\_t001 |  | \| \| |  |  |  |  |  |  |  |  |  |  |
| 1 | Vitvi19g04528\_t001 |  | \| \| |  |  |  |  |  |  |  |  |  |  |
| 1 | Vitvi19g02276\_t001 |  | \| \| |  |  |  |  |  |  |  |  |  |  |
| 1 | Vitvi19g04529\_t001 |  | \| \| |  |  |  |  |  |  |  |  |  |  |
| 1 | Vitvi19g01571\_t001 |  | Vitvi19g04559\_t001 |  |  |  |  |  |  |  |  |  |  |
| 1 | Vitvi19g04530\_t001 |  | \| \| |  |  |  |  |  |  |  |  |  |  |
| 1 | Vitvi19g04531\_t001 |  | \| \| |  |  |  |  |  |  |  |  |  |  |
| 1 | Vitvi19g04532\_t001 |  | \| \| |  |  |  |  |  |  |  |  |  |  |
| 1 | Vitvi19g04533\_t001 |  | \| \| |  |  |  |  |  |  |  |  |  |  |
| 1 | Vitvi19g04534\_t001 |  | \| \| |  |  |  |  |  |  |  |  |  |  |
| 1 | Vitvi19g04535\_t001 |  | \| \| |  |  |  |  |  |  |  |  |  |  |
| 1 | Vitvi19g04536\_t001 |  | \| \| |  |  |  |  |  |  |  |  |  |  |
| 1 | Vitvi19g04537\_t001 |  | \| \| |  |  |  |  |  |  |  |  |  |  |
| 1 | Vitvi19g04538\_t001 |  | \| \| |  |  |  |  |  |  |  |  |  |  |
| 1 | Vitvi19g04539\_t001 |  | \| \| |  |  |  |  |  |  |  |  |  |  |
| 1 | Vitvi19g04540\_t001 |  | \| \| |  |  |  |  |  |  |  |  |  |  |
| 1 | Vitvi19g04541\_t001 |  | \| \| |  |  |  |  |  |  |  |  |  |  |
| 1 | Vitvi19g04542\_t001 |  | \| \| |  |  |  |  |  |  |  |  |  |  |
| 1 | Vitvi19g04543\_t001 |  | \| \| |  |  |  |  |  |  |  |  |  |  |
| 1 | Vitvi19g01577\_t001 |  | Vitvi19g04558\_t001 |  |  |  |  |  |  |  |  |  |  |
| 1 | Vitvi19g04544\_t001 |  | \| \| |  |  |  |  |  |  |  |  |  |  |
| 1 | Vitvi19g01578\_t001 |  | Vitvi19g02279\_t001 |  |  |  |  |  |  |  |  |  |  |
| 0 | Vitvi19g02278\_t001 |  |  |  |  |  |  |  |  |  |  |  |  |
| 1 | Vitvi19g02279\_t001 |  | Vitvi19g01578\_t001 |  |  |  |  |  |  |  |  |  |  |
| 1 | Vitvi19g04545\_t001 |  | \| \| |  |  |  |  |  |  |  |  |  |  |
| 1 | Vitvi19g04546\_t001 |  | \| \| |  |  |  |  |  |  |  |  |  |  |
| 1 | Vitvi19g04547\_t001 |  | \| \| |  |  |  |  |  |  |  |  |  |  |
| 1 | Vitvi19g04548\_t001 |  | \| \| |  |  |  |  |  |  |  |  |  |  |
| 1 | Vitvi19g04549\_t001 |  | \| \| |  |  |  |  |  |  |  |  |  |  |
| 1 | Vitvi19g04550\_t001 |  | \| \| |  |  |  |  |  |  |  |  |  |  |
| 1 | Vitvi19g04551\_t001 |  | \| \| |  |  |  |  |  |  |  |  |  |  |
| 1 | Vitvi19g04552\_t001 |  | \| \| |  |  |  |  |  |  |  |  |  |  |
| 1 | Vitvi19g04553\_t001 |  | \| \| |  |  |  |  |  |  |  |  |  |  |
| 1 | Vitvi19g04554\_t001 |  | \| \| |  |  |  |  |  |  |  |  |  |  |
| 1 | Vitvi19g04555\_t001 |  | \| \| |  |  |  |  |  |  |  |  |  |  |
| 1 | Vitvi19g04556\_t001 |  | \| \| |  |  |  |  |  |  |  |  |  |  |
| 1 | Vitvi19g04557\_t001 |  | \| \| |  |  |  |  |  |  |  |  |  |  |
| 1 | Vitvi19g02282\_t001 |  | \| \| |  |  |  |  |  |  |  |  |  |  |
| 1 | Vitvi19g04558\_t001 |  | Vitvi19g01577\_t001 |  |  |  |  |  |  |  |  |  |  |
| 1 | Vitvi19g04559\_t001 |  | Vitvi19g01571\_t001 |  |  |  |  |  |  |  |  |  |  |
| 1 | Vitvi19g04560\_t001 |  | \| \| |  |  |  |  |  |  |  |  |  |  |
| 1 | Vitvi19g01585\_t001 |  | \| \| |  |  |  |  |  |  |  |  |  |  |
| 1 | Vitvi19g04561\_t001 |  | \| \| |  |  |  |  |  |  |  |  |  |  |
| 1 | Vitvi19g02286\_t001 |  | \| \| |  |  |  |  |  |  |  |  |  |  |
| 1 | Vitvi19g01586\_t001 |  | Vitvi19g01565\_t001 |  |  |  |  |  |  |  |  |  |  |
| 1 | Vitvi19g01588\_t001 |  | \| \| |  |  |  |  |  |  |  |  |  |  |
| 1 | Vitvi19g01589\_t001 |  | \| \| |  |  |  |  |  |  |  |  |  |  |
| 1 | Vitvi19g02287\_t001 |  | \| \| |  |  |  |  |  |  |  |  |  |  |
| 1 | Vitvi19g01592\_t001 |  | \| \| |  |  |  |  |  |  |  |  |  |  |
| 1 | Vitvi19g04562\_t001 |  | \| \| |  |  |  |  |  |  |  |  |  |  |
| 1 | Vitvi19g01596\_t001 |  | \| \| |  |  |  |  |  |  |  |  |  |  |
| 1 | Vitvi19g04563\_t001 |  | \| \| |  |  |  |  |  |  |  |  |  |  |
| 1 | Vitvi19g01601\_t001 |  | \| \| |  |  |  |  |  |  |  |  |  |  |
| 1 | Vitvi19g01602\_t001 |  | \| \| |  |  |  |  |  |  |  |  |  |  |
| 1 | Vitvi19g04564\_t001 |  | \| \| |  |  |  |  |  |  |  |  |  |  |
| 1 | Vitvi19g04565\_t001 |  | \| \| |  |  |  |  |  |  |  |  |  |  |
| 1 | Vitvi19g04566\_t001 |  | \| \| |  |  |  |  |  |  |  |  |  |  |
| 1 | Vitvi19g04567\_t001 |  | \| \| |  |  |  |  |  |  |  |  |  |  |
| 1 | Vitvi19g04568\_t001 |  | \| \| |  |  |  |  |  |  |  |  |  |  |
| 1 | Vitvi19g01606\_t001 |  | \| \| |  |  |  |  |  |  |  |  |  |  |
| 1 | Vitvi19g04569\_t001 |  | \| \| |  |  |  |  |  |  |  |  |  |  |
| 1 | Vitvi19g04570\_t001 |  | \| \| |  |  |  |  |  |  |  |  |  |  |
| 1 | Vitvi19g01607\_t001 |  | \| \| |  |  |  |  |  |  |  |  |  |  |
| 1 | Vitvi19g02292\_t001 |  | \| \| |  |  |  |  |  |  |  |  |  |  |
| 1 | Vitvi19g04571\_t001 |  | \| \| |  |  |  |  |  |  |  |  |  |  |
| 1 | Vitvi19g02295\_t001 |  | \| \| |  |  |  |  |  |  |  |  |  |  |
| 1 | Vitvi19g01610\_t001 |  | Vitvi19g02270\_t001 |  |  |  |  |  |  |  |  |  |  |
| 1 | Vitvi19g04572\_t001 |  | \| \| |  |  |  |  |  |  |  |  |  |  |
| 1 | Vitvi19g01612\_t001 |  | \| \| |  |  |  |  |  |  |  |  |  |  |
| 1 | Vitvi19g04573\_t001 |  | \| \| |  |  |  |  |  |  |  |  |  |  |
| 1 | Vitvi19g04574\_t001 |  | \| \| |  |  |  |  |  |  |  |  |  |  |
| 1 | Vitvi19g01614\_t001 |  | \| \| |  |  |  |  |  |  |  |  |  |  |
| 1 | Vitvi19g01616\_t001 |  | \| \| |  |  |  |  |  |  |  |  |  |  |
| 1 | Vitvi19g04575\_t001 |  | \| \| |  |  |  |  |  |  |  |  |  |  |
| 1 | Vitvi19g02300\_t001 |  | Vitvi19g04517\_t001 |  |  |  |  |  |  |  |  |  |  |
| 1 | Vitvi19g02302\_t001 |  | \| \| |  |  |  |  |  |  |  |  |  |  |
| 1 | Vitvi19g02303\_t001 |  | \| \| |  |  |  |  |  |  |  |  |  |  |
| 1 | Vitvi19g04576\_t001 |  | \| \| |  |  |  |  |  |  |  |  |  |  |
| 1 | Vitvi19g04577\_t001 |  | \| \| |  |  |  |  |  |  |  |  |  |  |
| 1 | Vitvi19g04578\_t001 |  | \| \| |  |  |  |  |  |  |  |  |  |  |
| 1 | Vitvi19g04579\_t001 |  | \| \| |  |  |  |  |  |  |  |  |  |  |
| 1 | Vitvi19g04580\_t001 |  | \| \| |  |  |  |  |  |  |  |  |  |  |
| 1 | Vitvi19g04581\_t001 |  | Vitvi19g04508\_t001 |  |  |  |  |  |  |  |  |  |  |
| 0 | Vitvi19g01617\_t001 |  |  |  |  |  |  |  |  |  |  |  |  |
| 0 | Vitvi19g04582\_t001 |  |  |  |  |  |  |  |  |  |  |  |  |
| 0 | Vitvi19g04583\_t001 |  |  |  |  |  |  |  |  |  |  |  |  |
| 0 | Vitvi19g02308\_t001 |  |  |  |  |  |  |  |  |  |  |  |  |
| 0 | Vitvi19g04584\_t001 |  |  |  |  |  |  |  |  |  |  |  |  |
| 0 | Vitvi19g04585\_t001 |  |  |  |  |  |  |  |  |  |  |  |  |
| 0 | Vitvi19g04586\_t001 |  |  |  |  |  |  |  |  |  |  |  |  |
| 0 | Vitvi19g02309\_t001 |  |  |  |  |  |  |  |  |  |  |  |  |
| 0 | Vitvi19g01620\_t001 |  |  |  |  |  |  |  |  |  |  |  |  |
| 0 | Vitvi19g04587\_t001 |  |  |  |  |  |  |  |  |  |  |  |  |
| 0 | Vitvi19g01622\_t001 |  |  |  |  |  |  |  |  |  |  |  |  |
| 0 | Vitvi19g04588\_t001 |  |  |  |  |  |  |  |  |  |  |  |  |
| 0 | Vitvi19g01624\_t001 |  |  |  |  |  |  |  |  |  |  |  |  |
| 0 | Vitvi19g04589\_t001 |  |  |  |  |  |  |  |  |  |  |  |  |
| 0 | Vitvi19g04590\_t001 |  |  |  |  |  |  |  |  |  |  |  |  |
| 0 | Vitvi19g04591\_t001 |  |  |  |  |  |  |  |  |  |  |  |  |
| 0 | Vitvi19g04592\_t001 |  |  |  |  |  |  |  |  |  |  |  |  |
| 0 | Vitvi19g01628\_t001 |  |  |  |  |  |  |  |  |  |  |  |  |
| 0 | Vitvi19g01629\_t001 |  |  |  |  |  |  |  |  |  |  |  |  |
| 0 | Vitvi19g04593\_t001 |  |  |  |  |  |  |  |  |  |  |  |  |
| 0 | Vitvi19g01630\_t001 |  |  |  |  |  |  |  |  |  |  |  |  |
| 0 | Vitvi19g01631\_t001 |  |  |  |  |  |  |  |  |  |  |  |  |
| 0 | Vitvi19g04594\_t001 |  |  |  |  |  |  |  |  |  |  |  |  |
| 0 | Vitvi19g04595\_t001 |  |  |  |  |  |  |  |  |  |  |  |  |
| 0 | Vitvi19g01635\_t001 |  |  |  |  |  |  |  |  |  |  |  |  |
| 0 | Vitvi19g01636\_t001 |  |  |  |  |  |  |  |  |  |  |  |  |
| 0 | Vitvi19g04596\_t001 |  |  |  |  |  |  |  |  |  |  |  |  |
| 0 | Vitvi19g04597\_t001 |  |  |  |  |  |  |  |  |  |  |  |  |
| 0 | Vitvi19g01638\_t001 |  |  |  |  |  |  |  |  |  |  |  |  |
| 0 | Vitvi19g01640\_t002 |  |  |  |  |  |  |  |  |  |  |  |  |
| 0 | Vitvi19g01641\_t001 |  |  |  |  |  |  |  |  |  |  |  |  |
| 0 | Vitvi19g04598\_t001 |  |  |  |  |  |  |  |  |  |  |  |  |
| 0 | Vitvi19g04599\_t001 |  |  |  |  |  |  |  |  |  |  |  |  |
| 0 | Vitvi19g04600\_t001 |  |  |  |  |  |  |  |  |  |  |  |  |
| 0 | Vitvi19g01643\_t001 |  |  |  |  |  |  |  |  |  |  |  |  |
| 0 | Vitvi19g01644\_t001 |  |  |  |  |  |  |  |  |  |  |  |  |
| 0 | Vitvi19g04602\_t001 |  |  |  |  |  |  |  |  |  |  |  |  |
| 0 | Vitvi19g01648\_t001 |  |  |  |  |  |  |  |  |  |  |  |  |
| 0 | Vitvi19g01649\_t001 |  |  |  |  |  |  |  |  |  |  |  |  |
| 0 | Vitvi19g04603\_t001 |  |  |  |  |  |  |  |  |  |  |  |  |
| 0 | Vitvi19g01650\_t001 |  |  |  |  |  |  |  |  |  |  |  |  |
| 0 | Vitvi19g01651\_t001 |  |  |  |  |  |  |  |  |  |  |  |  |
| 0 | Vitvi19g01652\_t001 |  |  |  |  |  |  |  |  |  |  |  |  |
| 0 | Vitvi19g01654\_t001 |  |  |  |  |  |  |  |  |  |  |  |  |
| 0 | Vitvi19g01655\_t001 |  |  |  |  |  |  |  |  |  |  |  |  |
| 0 | Vitvi19g04604\_t001 |  |  |  |  |  |  |  |  |  |  |  |  |
| 0 | Vitvi19g02320\_t001 |  |  |  |  |  |  |  |  |  |  |  |  |
| 0 | Vitvi19g01657\_t001 |  |  |  |  |  |  |  |  |  |  |  |  |
| 0 | Vitvi19g01661\_t001 |  |  |  |  |  |  |  |  |  |  |  |  |
| 0 | Vitvi19g02321\_t001 |  |  |  |  |  |  |  |  |  |  |  |  |
| 0 | Vitvi19g02322\_t001 |  |  |  |  |  |  |  |  |  |  |  |  |
| 0 | Vitvi19g04605\_t001 |  |  |  |  |  |  |  |  |  |  |  |  |
| 0 | Vitvi19g02323\_t001 |  |  |  |  |  |  |  |  |  |  |  |  |
| 0 | Vitvi19g04606\_t001 |  |  |  |  |  |  |  |  |  |  |  |  |
| 0 | Vitvi19g02324\_t001 |  |  |  |  |  |  |  |  |  |  |  |  |
| 0 | Vitvi19g04607\_t001 |  |  |  |  |  |  |  |  |  |  |  |  |
| 0 | Vitvi19g01663\_t001 |  |  |  |  |  |  |  |  |  |  |  |  |
| 0 | Vitvi19g04608\_t001 |  |  |  |  |  |  |  |  |  |  |  |  |
| 0 | Vitvi19g01665\_t001 |  |  |  |  |  |  |  |  |  |  |  |  |
| 0 | Vitvi19g01666\_t001 |  |  |  |  |  |  |  |  |  |  |  |  |
| 0 | Vitvi19g01667\_t001 |  |  |  |  |  |  |  |  |  |  |  |  |
| 0 | Vitvi19g01668\_t003 |  |  |  |  |  |  |  |  |  |  |  |  |
| 0 | Vitvi19g01669\_t001 |  |  |  |  |  |  |  |  |  |  |  |  |
| 0 | Vitvi19g04609\_t001 |  |  |  |  |  |  |  |  |  |  |  |  |
| 0 | Vitvi19g01671\_t001 |  |  |  |  |  |  |  |  |  |  |  |  |
| 0 | Vitvi19g02328\_t001 |  |  |  |  |  |  |  |  |  |  |  |  |
| 0 | Vitvi19g01672\_t001 |  |  |  |  |  |  |  |  |  |  |  |  |
| 0 | Vitvi19g04610\_t001 |  |  |  |  |  |  |  |  |  |  |  |  |
| 0 | Vitvi19g01673\_t001 |  |  |  |  |  |  |  |  |  |  |  |  |
| 0 | Vitvi19g04611\_t001 |  |  |  |  |  |  |  |  |  |  |  |  |
| 0 | Vitvi19g04612\_t001 |  |  |  |  |  |  |  |  |  |  |  |  |
| 0 | Vitvi19g04613\_t001 |  |  |  |  |  |  |  |  |  |  |  |  |
| 0 | Vitvi19g04614\_t001 |  |  |  |  |  |  |  |  |  |  |  |  |
| 0 | Vitvi19g04615\_t001 |  |  |  |  |  |  |  |  |  |  |  |  |
| 0 | Vitvi19g01676\_t001 |  |  |  |  |  |  |  |  |  |  |  |  |
| 0 | Vitvi19g04616\_t001 |  |  |  |  |  |  |  |  |  |  |  |  |
| 0 | Vitvi19g02329\_t001 |  |  |  |  |  |  |  |  |  |  |  |  |
| 0 | Vitvi19g02331\_t001 |  |  |  |  |  |  |  |  |  |  |  |  |
| 0 | Vitvi19g01677\_t001 |  |  |  |  |  |  |  |  |  |  |  |  |
| 0 | Vitvi19g02333\_t001 |  |  |  |  |  |  |  |  |  |  |  |  |
| 0 | Vitvi19g02334\_t001 |  |  |  |  |  |  |  |  |  |  |  |  |
| 0 | Vitvi19g02335\_t001 |  |  |  |  |  |  |  |  |  |  |  |  |
| 0 | Vitvi19g02336\_t001 |  |  |  |  |  |  |  |  |  |  |  |  |
| 0 | Vitvi19g01678\_t001 |  |  |  |  |  |  |  |  |  |  |  |  |
| 0 | Vitvi19g01679\_t001 |  |  |  |  |  |  |  |  |  |  |  |  |
| 0 | Vitvi19g04617\_t001 |  |  |  |  |  |  |  |  |  |  |  |  |
| 0 | Vitvi19g01680\_t001 |  |  |  |  |  |  |  |  |  |  |  |  |
| 0 | Vitvi19g04618\_t001 |  |  |  |  |  |  |  |  |  |  |  |  |
| 0 | Vitvi19g01681\_t001 |  |  |  |  |  |  |  |  |  |  |  |  |
| 0 | Vitvi19g04619\_t001 |  |  |  |  |  |  |  |  |  |  |  |  |
| 0 | Vitvi19g01685\_t001 |  |  |  |  |  |  |  |  |  |  |  |  |
| 0 | Vitvi19g01686\_t001 |  |  |  |  |  |  |  |  |  |  |  |  |
| 0 | Vitvi19g01687\_t001 |  |  |  |  |  |  |  |  |  |  |  |  |
| 0 | Vitvi19g04620\_t001 |  |  |  |  |  |  |  |  |  |  |  |  |
| 0 | Vitvi19g01688\_t001 |  |  |  |  |  |  |  |  |  |  |  |  |
| 0 | Vitvi19g01690\_t001 |  |  |  |  |  |  |  |  |  |  |  |  |
| 0 | Vitvi19g04621\_t001 |  |  |  |  |  |  |  |  |  |  |  |  |
| 0 | Vitvi19g04622\_t001 |  |  |  |  |  |  |  |  |  |  |  |  |
| 0 | Vitvi19g01692\_t001 |  |  |  |  |  |  |  |  |  |  |  |  |
| 0 | Vitvi19g01695\_t002 |  |  |  |  |  |  |  |  |  |  |  |  |
| 0 | Vitvi19g04623\_t001 |  |  |  |  |  |  |  |  |  |  |  |  |
| 0 | Vitvi19g01697\_t001 |  |  |  |  |  |  |  |  |  |  |  |  |
| 0 | Vitvi19g04624\_t001 |  |  |  |  |  |  |  |  |  |  |  |  |
| 0 | Vitvi19g04625\_t001 |  |  |  |  |  |  |  |  |  |  |  |  |
| 0 | Vitvi19g01702\_t001 |  |  |  |  |  |  |  |  |  |  |  |  |
| 0 | Vitvi19g01703\_t001 |  |  |  |  |  |  |  |  |  |  |  |  |
| 0 | Vitvi19g01704\_t001 |  |  |  |  |  |  |  |  |  |  |  |  |
| 0 | Vitvi19g01705\_t002 |  |  |  |  |  |  |  |  |  |  |  |  |
| 0 | Vitvi19g01706\_t001 |  |  |  |  |  |  |  |  |  |  |  |  |
| 0 | Vitvi19g01707\_t001 |  |  |  |  |  |  |  |  |  |  |  |  |
| 0 | Vitvi19g04626\_t001 |  |  |  |  |  |  |  |  |  |  |  |  |
| 0 | Vitvi19g01708\_t001 |  |  |  |  |  |  |  |  |  |  |  |  |
| 0 | Vitvi19g04627\_t001 |  |  |  |  |  |  |  |  |  |  |  |  |
| 0 | Vitvi19g04628\_t001 |  |  |  |  |  |  |  |  |  |  |  |  |
| 0 | Vitvi19g02343\_t001 |  |  |  |  |  |  |  |  |  |  |  |  |
| 0 | Vitvi19g01710\_t001 |  |  |  |  |  |  |  |  |  |  |  |  |
| 0 | Vitvi19g02346\_t001 |  |  |  |  |  |  |  |  |  |  |  |  |
| 0 | Vitvi19g02347\_t001 |  |  |  |  |  |  |  |  |  |  |  |  |
| 0 | Vitvi19g01711\_t001 |  |  |  |  |  |  |  |  |  |  |  |  |
| 0 | Vitvi19g01712\_t001 |  |  |  |  |  |  |  |  |  |  |  |  |
| 0 | Vitvi19g04629\_t001 |  |  |  |  |  |  |  |  |  |  |  |  |
| 0 | Vitvi19g01715\_t001 |  |  |  |  |  |  |  |  |  |  |  |  |
| 0 | Vitvi19g04630\_t001 |  |  |  |  |  |  |  |  |  |  |  |  |
| 0 | Vitvi19g01718\_t001 |  |  |  |  |  |  |  |  |  |  |  |  |
| 0 | Vitvi19g01719\_t001 |  |  |  |  |  |  |  |  |  |  |  |  |
| 0 | Vitvi19g01720\_t001 |  |  |  |  |  |  |  |  |  |  |  |  |
| 0 | Vitvi19g04631\_t001 |  |  |  |  |  |  |  |  |  |  |  |  |
| 0 | Vitvi19g04632\_t001 |  |  |  |  |  |  |  |  |  |  |  |  |
| 0 | Vitvi19g01724\_t001 |  |  |  |  |  |  |  |  |  |  |  |  |
| 0 | Vitvi19g01725\_t001 |  |  |  |  |  |  |  |  |  |  |  |  |
| 0 | Vitvi19g01726\_t001 |  |  |  |  |  |  |  |  |  |  |  |  |
| 0 | Vitvi19g01727\_t001 |  |  |  |  |  |  |  |  |  |  |  |  |
| 0 | Vitvi19g02351\_t001 |  |  |  |  |  |  |  |  |  |  |  |  |
| 0 | Vitvi19g04633\_t001 |  |  |  |  |  |  |  |  |  |  |  |  |
| 0 | Vitvi19g04634\_t001 |  |  |  |  |  |  |  |  |  |  |  |  |
| 0 | Vitvi19g01729\_t001 |  |  |  |  |  |  |  |  |  |  |  |  |
| 0 | Vitvi19g04635\_t001 |  |  |  |  |  |  |  |  |  |  |  |  |
| 0 | Vitvi19g04636\_t001 |  |  |  |  |  |  |  |  |  |  |  |  |
| 0 | Vitvi19g04637\_t001 |  |  |  |  |  |  |  |  |  |  |  |  |
| 0 | Vitvi19g01732\_t001 |  |  |  |  |  |  |  |  |  |  |  |  |
| 0 | Vitvi19g01733\_t001 |  |  |  |  |  |  |  |  |  |  |  |  |
| 0 | Vitvi19g01734\_t002 |  |  |  |  |  |  |  |  |  |  |  |  |
| 0 | Vitvi19g01737\_t001 |  |  |  |  |  |  |  |  |  |  |  |  |
| 0 | Vitvi19g01738\_t002 |  |  |  |  |  |  |  |  |  |  |  |  |
| 0 | Vitvi19g04638\_t001 |  |  |  |  |  |  |  |  |  |  |  |  |
| 0 | Vitvi19g01739\_t001 |  |  |  |  |  |  |  |  |  |  |  |  |
| 0 | Vitvi19g01741\_t001 |  |  |  |  |  |  |  |  |  |  |  |  |
| 0 | Vitvi19g01742\_t002 |  |  |  |  |  |  |  |  |  |  |  |  |
| 0 | Vitvi19g01743\_t001 |  |  |  |  |  |  |  |  |  |  |  |  |
| 0 | Vitvi19g01744\_t001 |  |  |  |  |  |  |  |  |  |  |  |  |
| 0 | Vitvi19g01746\_t001 |  |  |  |  |  |  |  |  |  |  |  |  |
| 0 | Vitvi19g01748\_t001 |  |  |  |  |  |  |  |  |  |  |  |  |
| 0 | Vitvi19g01747\_t001 |  |  |  |  |  |  |  |  |  |  |  |  |
| 0 | Vitvi19g01749\_t001 |  |  |  |  |  |  |  |  |  |  |  |  |
| 0 | Vitvi19g01751\_t001 |  |  |  |  |  |  |  |  |  |  |  |  |
| 0 | Vitvi19g04639\_t001 |  |  |  |  |  |  |  |  |  |  |  |  |
| 0 | Vitvi19g04640\_t001 |  |  |  |  |  |  |  |  |  |  |  |  |
| 0 | Vitvi19g04641\_t001 |  |  |  |  |  |  |  |  |  |  |  |  |
| 0 | Vitvi19g01754\_t001 |  |  |  |  |  |  |  |  |  |  |  |  |
| 0 | Vitvi19g01756\_t001 |  |  |  |  |  |  |  |  |  |  |  |  |
| 0 | Vitvi19g02355\_t001 |  |  |  |  |  |  |  |  |  |  |  |  |
| 0 | Vitvi19g01759\_t001 |  |  |  |  |  |  |  |  |  |  |  |  |
| 0 | Vitvi19g01760\_t001 |  |  |  |  |  |  |  |  |  |  |  |  |
| 0 | Vitvi19g01761\_t001 |  |  |  |  |  |  |  |  |  |  |  |  |
| 0 | Vitvi19g04642\_t001 |  |  |  |  |  |  |  |  |  |  |  |  |
| 0 | Vitvi19g04643\_t001 |  |  |  |  |  |  |  |  |  |  |  |  |
| 0 | Vitvi19g01763\_t001 |  |  |  |  |  |  |  |  |  |  |  |  |
| 0 | Vitvi19g01764\_t001 |  |  |  |  |  |  |  |  |  |  |  |  |
| 0 | Vitvi19g01767\_t001 |  |  |  |  |  |  |  |  |  |  |  |  |
| 0 | Vitvi19g01768\_t001 |  |  |  |  |  |  |  |  |  |  |  |  |
| 0 | Vitvi19g01769\_t001 |  |  |  |  |  |  |  |  |  |  |  |  |
| 0 | Vitvi19g01770\_t001 |  |  |  |  |  |  |  |  |  |  |  |  |
| 0 | Vitvi19g01771\_t001 |  |  |  |  |  |  |  |  |  |  |  |  |
| 0 | Vitvi19g01772\_t001 |  |  |  |  |  |  |  |  |  |  |  |  |
| 0 | Vitvi19g02356\_t001 |  |  |  |  |  |  |  |  |  |  |  |  |
| 0 | Vitvi19g01773\_t001 |  |  |  |  |  |  |  |  |  |  |  |  |
| 0 | Vitvi19g01774\_t001 |  |  |  |  |  |  |  |  |  |  |  |  |
| 0 | Vitvi19g04644\_t001 |  |  |  |  |  |  |  |  |  |  |  |  |
| 0 | Vitvi19g01776\_t001 |  |  |  |  |  |  |  |  |  |  |  |  |
| 0 | Vitvi19g01777\_t001 |  |  |  |  |  |  |  |  |  |  |  |  |
| 0 | Vitvi19g04645\_t001 |  |  |  |  |  |  |  |  |  |  |  |  |
| 0 | Vitvi19g01778\_t001 |  |  |  |  |  |  |  |  |  |  |  |  |
| 0 | Vitvi19g01783\_t001 |  |  |  |  |  |  |  |  |  |  |  |  |
